# *In situ* synthesis of ultrafine Cu(ii) metal immobilized on pectin hydrogel, modified by a CoFe_2_O_4_/Pr-SO_3_H nanocomposite as a green catalyst for reduction of nitro compounds and synthesis of 1*H*-tetrazoles

**DOI:** 10.1039/d4ra08706b

**Published:** 2025-01-15

**Authors:** Roya Mozafari, Maria Mohammadi, Setareh Moradi, Mohammad Ghadermazi

**Affiliations:** a Department of Chemistry, University of Kurdistan P. O. Box 66135-416 Sanandaj Iran mghadermazi@yahoo.com +98 873324133 +98 8733624133

## Abstract

Synthesis of 5-substituted 1*H*-tetrazoles and reduction of a variety of nitro compounds presents a promising solution for the pharmaceutical and agricultural industries. However, the development of green catalysts with superior catalytic performance for this reaction remains a significant challenge. This research introduces a green protocol for the *in situ* creation of ultrafine Cu(ii) metal immobilized on the surface of pectin hydrogel (HPEC), modified by a CoFe_2_O_4_/Pr-SO_3_H magnetic nanocomposite, enabling the synthesis of tetrazoles and reduction of nitro compounds. This catalyst exhibits superior catalytic performance under green reaction conditions, short reaction time, catalyst separation, and thermal stability. The heterogeneous catalyst's structure and composition were thoroughly analyzed using various techniques such as FT-IR, FE-SEM, VSM, ICP-OES, TGA, XRD, BET, EDX, and X-ray mapping.

## Abstract 2

This research introduced CoFe_2_O_4_@HPECG/Pr-SO_3_H·Cu(ii) MNPs as green catalyst for the aqueous reduction of nitro compounds to amines and synthesis of 1*H*-tetrazoles.

## Graphical abstract

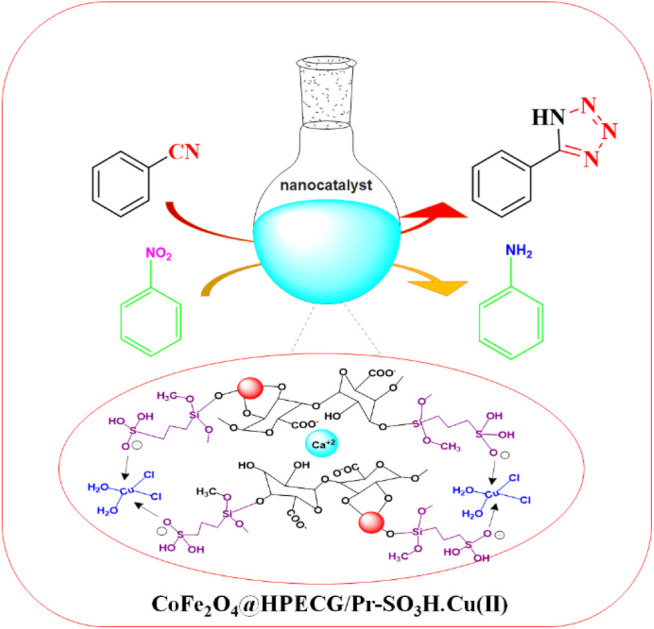

## 1. Introduction

Green chemistry is an innovative field that aims to foster sustainability at the molecular level by optimizing the use of renewable raw materials and eliminating destructive and potentially harmful reagents and solvents in chemical product creation and application.^1–8^

Hydrogels are three-dimensional polymer networks that possess cross-links and the capacity to absorb significant quantities of water or biological fluids, even under pressure.^9–11^ Natural hydrogels, due to their abundance, diversity, renewability, low cost, biodegradability, low toxicity, and biocompatibility, are particularly intriguing when compared to synthetic hydrogels.^12,13^ These unique properties make hydrogels desirable in various industries, such as food, packaging, medicine, agriculture, and even for absorbing contaminants.^14,15^

Plant cell walls consist of polysaccharides and proteins, where the polysaccharides are typically made up of pectin and cellulose.^16^ Pectin, comprising galacturonic acid-type substances, contains methyl esters and sodium and potassium salts.^17^ Pectin can be sourced from a variety of plants, such as apple pomace, tomatoes, sugarcane, lemons, kiwis, onions, garlic, orange peel, cacao, soybeans, and sunflower seeds.^18^ The diverse structures within pectin influence hydrogel formation in different ways.^19,20^ One potential application of pectin is as a substrate for catalysts.^21^

The development and creation of green catalysts have drawn significant interest in both academic and industrial sectors.^22,23^ To achieve this goal, making the catalysts heterogeneous on solid supports is one of the intriguing options to avoid catalyst waste.^24^ Moreover, the supports often significantly influence the catalyst's activity.^25,26^ The diversity of catalysts with solid bases can offer chances for the straightforward separation of catalysts from reaction mixtures.^27^ Indeed, separable magnetic catalysts are a better strategy to bridge the gap between homogeneous and heterogeneous catalysts.^28–30^

Nitroaromatic compounds are very toxic and are introduced into the environment through agricultural chemicals, paint wastewater, plastic, synthetic resins, *etc.*^31^ Conversely, the reduction of nitroarenes to arylamines yields important starting materials and intermediates widely used in the pharmaceutical and agricultural industries.^32^ Hence, the reduction of nitro compounds to amine groups using green solvents in a sustainable method has received significant attention.^33^ However, while the methods reported to date boast many advantages, they also come with a set of challenges.^34^ These include suboptimal efficiency, the employment of dangerous reagents, poor recovery of pricey catalysts, and extended reaction durations, resulting in the production of significant amounts of harmful waste.^35^ Therefore, there is a pressing need for more efficient, green, and sustainable methods.

Tetrazoles are non-cyclic compounds featuring a five-membered ring comprised of one carbon atom and four nitrogen atoms.^36^ These compounds have various uses in the creation of organic materials, in biological contexts, and within the pharmaceutical industry. The formation of tetrazole ring is an essential process in medicinal and organic chemistry, and various methods have been proposed for the synthesis of these compounds.^37^ Although numerous studies have explored the utilization of catalysts for the direct synthesis of tetrazole, there has been no paper dedicated to the application of CoFe_2_O_4_@HPECG/Pr-SO_3_H·Cu(ii) based heterogeneous catalysts in tetrazole synthesis. Consequently, it is highly preferred to develop an effective catalyst for the selective production of tetrazole under mild reaction conditions in an environmentally friendly solvent.

In this study, we present the first instance of creating a novel, magnetically retrievable nanocomposite consisting of CoFe_2_O_4_@HPECG/Pr-SO_3_H and Cu. This nanocomposite was created, analyzed, and tested as a catalyst for the reduction of nitro groups into amines using NaBH_4_ and synthesis of tetrazole in an aqueous medium. The newly synthesized nanocatalyst combines the catalytic prowess of Cu nanoparticles with the magnetic properties of CoFe_2_O_4_, showing remarkable catalytic activity.

## 2. Experimental

### 2.1. Materials

All the chemicals and solvents used for the synthesis of CoFe_2_O_4_@HPECG/Pr-SO_3_H·Cu(ii), including FeCl_3_·6H_2_O, CoCl_2_·6H_2_O, CuCl_2_·2H_2_O, NaBH_4_, HCl (37%), NaOH, NaN_3_ and EtOH (95%) and other chemicals were purchased from Merck and Sigma-Aldrich.

### 2.2. Characterization methods

The instruments used for the characterization of CoFe_2_O_4_@HPECG/Pr-SO_3_H·Cu(ii) included FT-IR, VSM, FE-SEM, TGA, BET, EDX, ICP-OES, XRD, and X-ray mapping. SEM-TESCAN MIRA3 was used to analyze the size and structure of the nanocatalyst. The samples were analyzed using XRD and FTIR techniques with JEOl JEM-1010 and JEOL JSM-6100 microscopes, respectively, in the 2*θ* = 20°–80° region and PerkinElmer Spectrum one instruments were used potassium bromide discs. Moreover, elemental analysis of all samples and thermogravimetric analysis (TGA) was determined by energy-dispersive X-ray spectroscopy (EDX) by a Kevex, Delta Class I and Shimadzu DTG-60 instrument respectively. A measurement of Vibrating Sample Magnetometer (VSM) was taken using a Vibrating Sample Magnetometer MDKFD. The pore size distribution and surface area were investigated using Barrett–Joyner–Halenda (BJH) analysis and Brunauer–Emmett–Teller (BET) measurements, respectively. The purity determination of the products and the reactions were monitored using TLC on silica gel Polygram SILG/UV 254 plates. Cu loadings in the CoFe_2_O_4_@HPECG/Pr-SO_3_H·Cu(ii) catalyst were determined by ICP-OES (730-ES Varian).

### 2.3. Synthesis of magnetic cobalt ferrite nanoparticles

In a 100 mL round-bottom flask, a mixture of 4.04 g (4 mmol) of Fe(NO_3_)_3_·6H_2_O and 1.45 g (2 mmol) of Co(NO_3_)_2_·6H_2_O was dissolved in 50 mL of distilled water. Then, a NaOH (3 M) solution was slowly added to the mixture with vigorous stirring and heated at 80 °C for 1 h. This will facilitate the precipitation of the cobalt ferrite nanoparticles. The produced magnetic nanoparticles were separated using an external magnet and were washed several times with ethanol and water. The washed nanoparticles were transferred to the oven and dried at 50 °C for 30 min.^38^

### 2.4. Pectin extraction from green lemon and its purification

5 g of ground lemon peel was placed in a 250 mL beaker, and 150 mL of distilled water was added. The mixture was stirred using a magnetic stirrer for 30 min at room temperature. Then, 10 mL of concentrated HCl was slowly added to the reaction mixture, which was then stirred at 75 °C for 85 min. This process will help break down the cell walls and release the pectin. Using filter paper, the precipitate was separated and an equal volume of ethanol (95%) was added to the resulting solution, causing the pectin to float on the surface in a suspension form. The light brown jelly-like portion was separated using a centrifuge and, after being washed with acetone, was dried in an oven at 25 °C for 48 h. The dried jellies turned into a soft powder, from which 0.44 g of pure pectin (PEC) was extracted and characterized by FT-IR spectroscopy.

### 2.5. Acidification of pectin

This method is to modify pectin by making it alkaline and then acidifying it. The purpose of such treatment is often to modify the properties of pectin to make it more suitable for specific applications or to obtain a specific derivative of pectin. In a 100 mL beaker, 0.44 g of the extracted pectin was dissolved in 44 mL of distilled water, resulting in a solution with a pH of 3.2.4 g of NaOH was added to 20 mL of distilled water, and this second solution was added dropwise to the pectin solution under vigorous stirring in an ice bath until the pH reached 12.2. After stirring for an hour, the mixture was left at 40 °C for 24 h. Afterwards, the pH of the resulting solution was measured (pH = 11.5). A few drops of 37% HCl were added until the pH reached 4.8. After adding an equal volume of ethanol, the precipitate was separated by centrifugation. The resulting product (HPEC) was dried at room temperature for 24 h.

### 2.6. Preparation of pectin hydrogel

0.3 g of acid-hydrolyzed pectin were added to 100 mL of distilled water and stirred until dissolved.

0.4 g of calcium chloride were dissolved in 70 mL of distilled water and added dropwise to the pectin solution under vigorous stirring. The calcium ions will act as crosslinking agents, forming a hydrogel structure with pectin. 15 mL of ethanol (1 M) and 25 mL of methanol were then added to the resulting solution. This will promote the precipitation of the pectin hydrogel. The pectin hydrogel precipitate (HPECG) was separated by centrifugation. After being washed twice with ethanol to remove any residual reagents or impurities, the precipitate was dried at 25 °C for 24 h.

### 2.7. Preparation of CoFe_2_O_4_@HPEC nanoparticles

0.36 g of synthesized HPEC was mixed with 72 mL of distilled water at 70 °C with vigorous stirring until the HPEC is fully dissolved. 0.36 g of cobalt ferrite were dissolved in 72 mL of distilled water, sonicated for 20 min to ensure uniform dispersion and reduce any agglomerates, then dropwise added to the pectin solution, followed by an additional 20 min of sonication. The mixture was then allowed to sit at 70 °C for 24 h. After which the resulting precipitate was separated using a magnet and then washed with water and ethanol. The residue was dried at 50 °C for 30 min.

### 2.8. Preparation of CoFe_2_O_4_@HPECG/Pr-SO_3_H

In a 100 mL round-bottom flask, 5.0 g of CoFe_2_O_4_@HPECG were mixed with 25 mL of methanol and 1 mL of 3-trimethoxysilyl-1-propanethiol (MPTMS). The mixture was stirred with a magnetic stirrer at room temperature for 24 h. The resulting product was separated using an external magnet and washed with distilled water. The synthesized CoFe_2_O_4_@HPECG/Pr-SO_3_H was dried at 50 °C. The obtained product was dissolved in 10 mL of distilled water, and dropwise, over the course of an hour, 10 mL of H_2_O_2_ (30%) was added to it. This step will oxidize the thiol groups to sulfonic acid groups. The mixture was stirred with a magnetic stirrer for 16 h. CoFe_2_O_4_@HPECG/Pr-SO_3_H was separated, washed with distilled water, and then dried.

### 2.9. Preparation of CoFe_2_O_4_@HPECG/Pr-SO_3_H·Cu(ii)

In a 50 mL flask, 2.0 g of the nanocomposite CoFe_2_O_4_@HPECG/Pr-SO_3_H was dissolved in 8 mL of distilled water and sonicated for 20 min to ensure the nanocomposite is well-dispersed. Then, 16.0 g of CuCl_2_·2H_2_O were dissolved in 12 mL of distilled water and added to the nanocomposite solution. The mixture was stirred with a magnetic stirrer for 12 h. Then, the obtained product was washed with distilled water to remove any unbound Cu(ii) ions or other impurities.

### 2.10. A common method to reduce nitro compounds with NaBH_4_ in the presence of CoFe_2_O_4_@HPECG/Pr-SO_3_H·Cu(ii) nanocatalyst

A combination of 25 mg CoFe_2_O_4_@HPECG/Pr-SO_3_H·Cu(ii) in 4 mL of distilled water and 0.015 mmol NaBH_4_ was prepared in a round-bottomed flask that had a magnetic stirrer. After 3 min, 1 mmol of nitro compound was added to the flask and the mixture was vigorously stirred at ambient temperature. The progress of the reaction was used by TLC. After the reaction completed, an external magnet was used to easily separate the magnetic nanocatalyst from the reaction mixture. Next, the reduced product was extracted from the remaining liquid by utilizing diethyl ether.

### 2.11. General process for the synthesis of 1*H*-tetrazoles

A combination of 1 mmol nitrile, 1.2 mmol sodium azide and 40 mg CoFe_2_O_4_@HPECG/Pr-SO_3_H·Cu(ii) in 3 mL of distilled water was prepared in a round-bottomed flask equipped with a magnetic stirred at 70 °C. The progress of the reaction was used by TLC. After the reaction completed, an external magnet was used to easily separate the magnetic nanocatalyst from the reaction mixture. The mixture was subsequently treated with ethyl acetate and HCl while being stirred vigorously. The organic layer was removed from the mixture, and the aqueous layer was extracted with ethyl acetate. Ultimately, the separated organic layers underwent a wash with water, after which the solvent was eliminated, and crystallization in ethanol for further purification.

## 3. Results and discussion

### 3.1. Characterization of CoFe_2_O_4_@HPECG/Pr-SO_3_H·Cu(ii) nanocatalyst

**Scheme 1:**
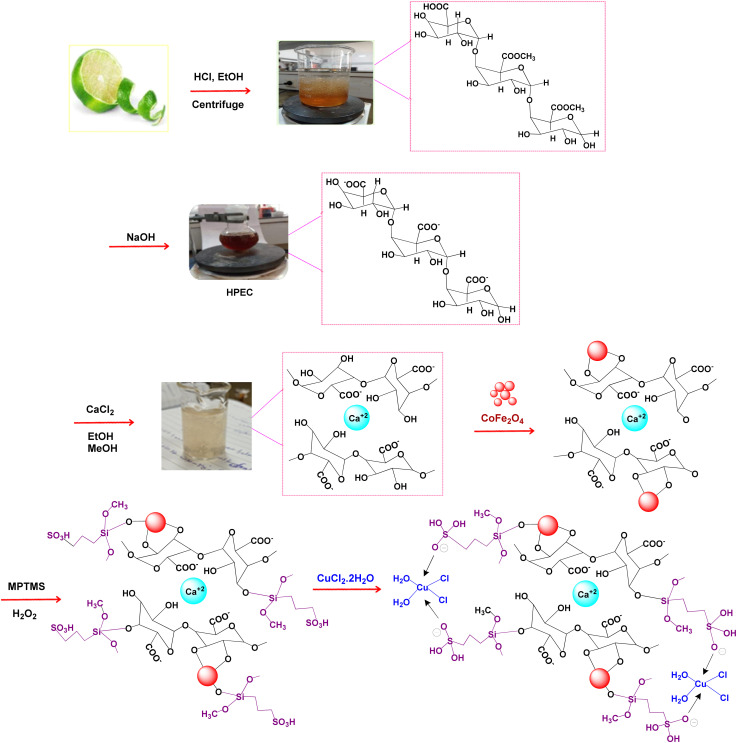
Synthesis CoFe_2_O_4_@HPECG/Pr-SO_3_H·Cu(ii).

The synthetic strategy for the preparation of the CoFe_2_O_4_@HPECG/Pr-SO_3_H·Cu(ii) is summarized in Scheme 1. First, pectin, as an important biopolymer, is extracted from lemon peel and alkali de-esterified. The purpose of such treatment is often to modify the properties of pectin to make it more suitable for specific applications or to obtain a specific derivative of pectin. Pectin hydrogel was prepared by ionically cross-linking of pectin solution in the presence of calcium chloride. Next, CoFe_2_O_4_ magnetic nanoparticles are deposited onto the surface of this pectin hydrogels. This step allows for proper embedding of the magnetic nanoparticles within the pectin matrix. At the next step, by adding MPTMS and subsequent oxidation with H_2_O_2_ was sulfonated (CoFe_2_O_4_@HPECG/Pr-SO_3_H). Finally, CoFe_2_O_4_@HPECG/Pr-SO_3_H was treated with CuCl_2_·2H_2_O to give CoFe_2_O_4_@HPECG/Pr-SO_3_H·Cu(ii). This allows the Cu(ii) ions to bind with the sulfonic acid groups on the nanocomposite, forming the desired CoFe_2_O_4_@HPECG/Pr-SO_3_H·Cu(ii) product.

#### 3.1.1. FE-SEM studies

**Fig. 1:**
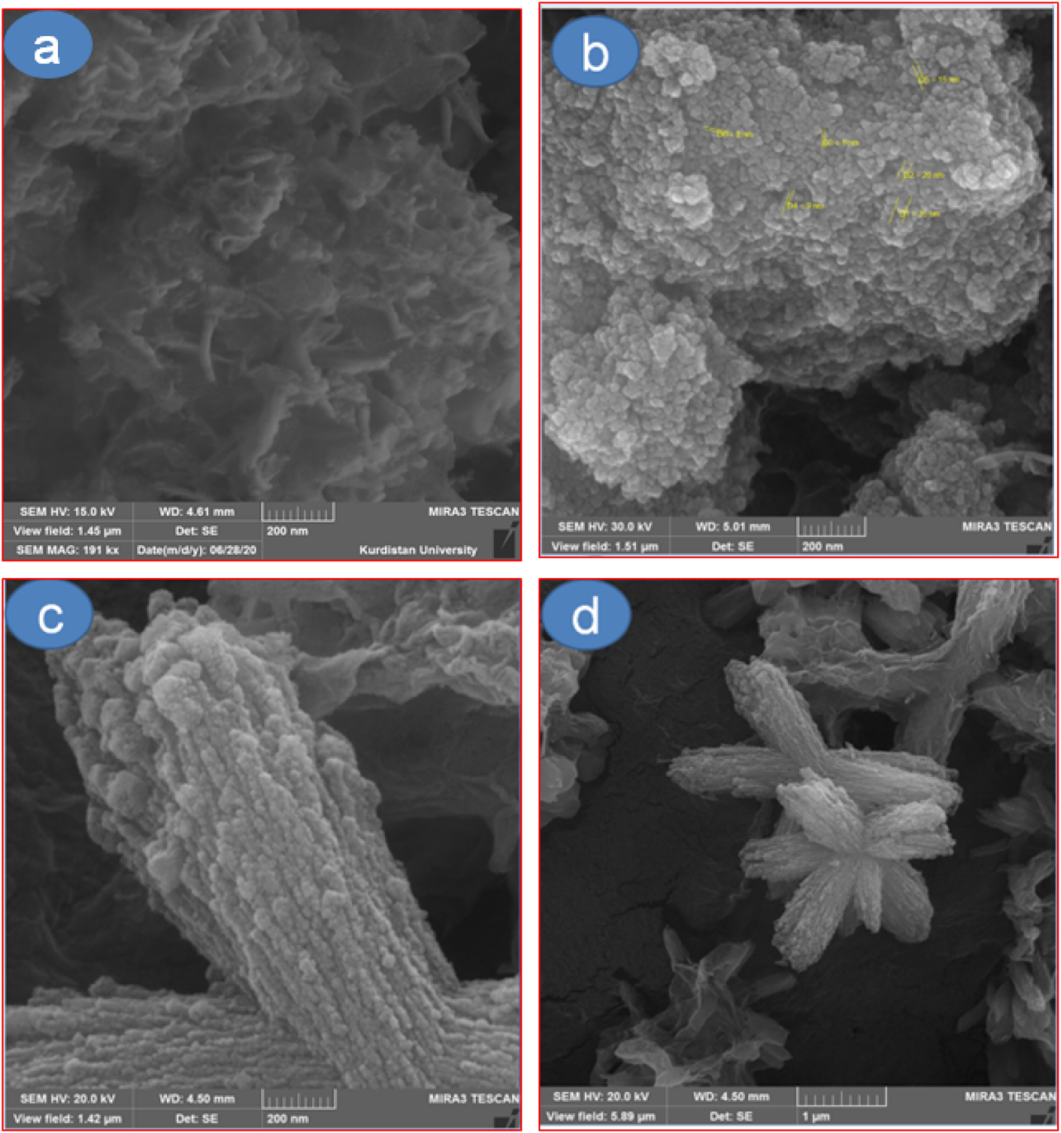
Field emission scanning electron microscopy (FE-SEM) images of HPECG (a), CoFe_2_O_4_ (b) and CoFe_2_O_4_@HPECG/Pr-SO_3_H·Cu(ii) nanocatalyst at 200 nm (c), and 1 μm, (d) magnifications.

The morphology and particle size of CoFe_2_O_4_@HPECG/Pr-SO_3_H·Cu(ii) were determined using the SEM technique. The SEM images of HPECG (a) CoFe_2_O_4_ (b) and CoFe_2_O_4_@HPECG/Pr-SO_3_H·Cu(ii) (c-d) at different magnifications are shown in Fig. 1. The results obtained from image (a) show that the structure is amorphous. Image (b) demonstrates that the CoFe_2_O_4_ structure is in the form of nanospheres with an average size of 14 nm. Also, upon analyzing these images (c and d), it was determined that the synthesized sample is rod-shaped and can be seen as star-shaped from a distance. The average size of the nanocatalyst is 28 nm, and due to the electronic and magnetic interaction between nanoparticles, they are somewhat connected to each other. Furthermore, the distribution of nanoparticles throughout the substrate is completely uniform, indicating the successful formation of the CoFe_2_O_4_@HPECG/Pr-SO_3_H·Cu(ii).

#### 3.1.2. EDX and elemental mapping analysis

The element composition of the synthesized CoFe_2_O_4_@HPECG/Pr-SO_3_H·Cu(ii) was studied using EDX and elemental mapping analysis which showed the existence of Co, Fe, S, O, C, Ca, Si, and Cu in the prepared nanocatalyst. The results confirmed the successful immobilization mentioned Pr-SO_3_H·Cu(ii) catalytic complex on the surface of CoFe_2_O_4_@HPECG nanoparticles.

**Fig. 2:**
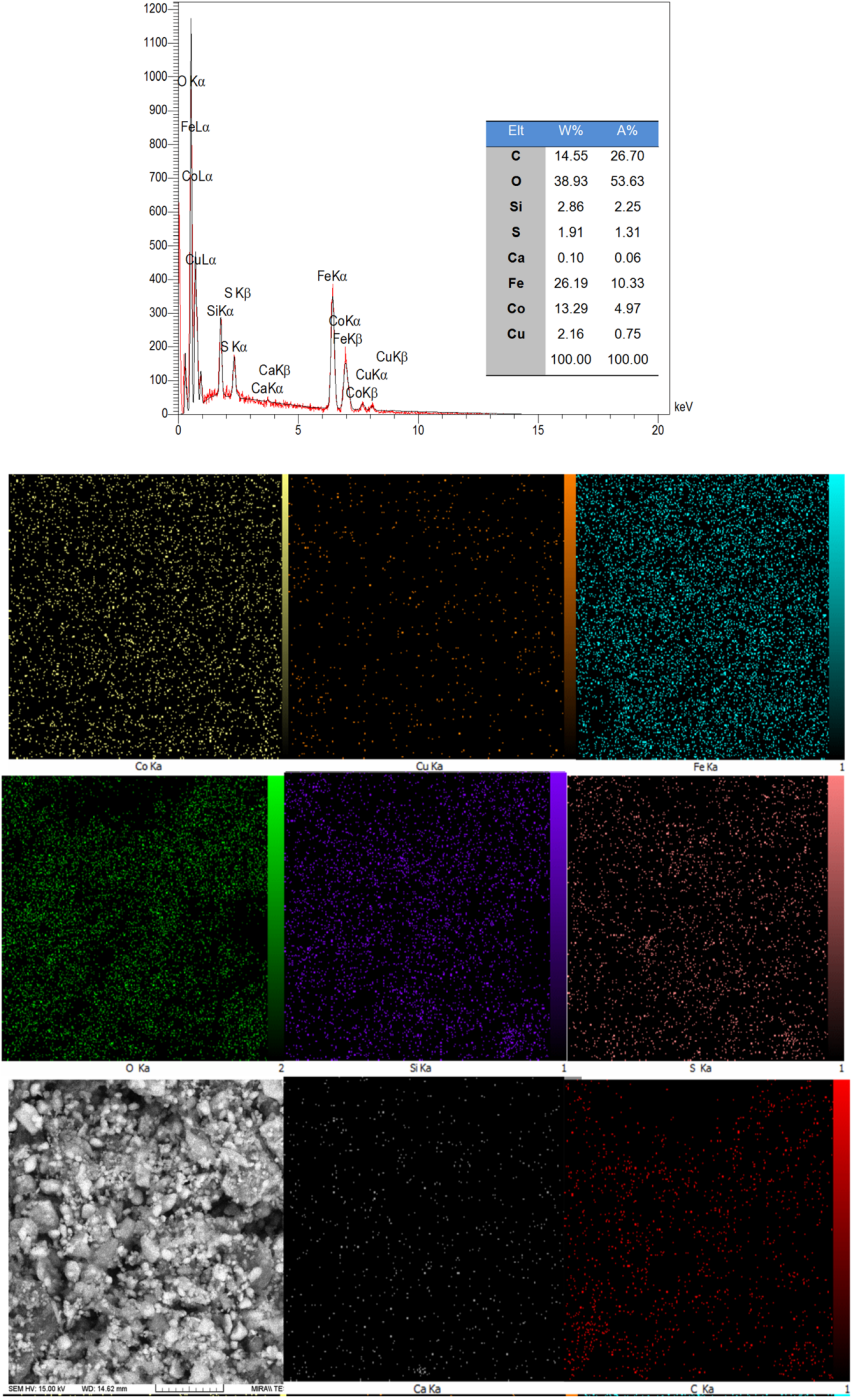
EDX spectrum and elemental mapping CoFe_2_O_4_@HPECG/Pr-SO_3_H·Cu(ii) nanocomposites.

Additionally, the elemental mapping results indicate that these elements are evenly spread out across the HPECG, indicating an integrated system (Fig. 2).

#### 3.1.3. VSM analysis studies

**Fig. 3:**
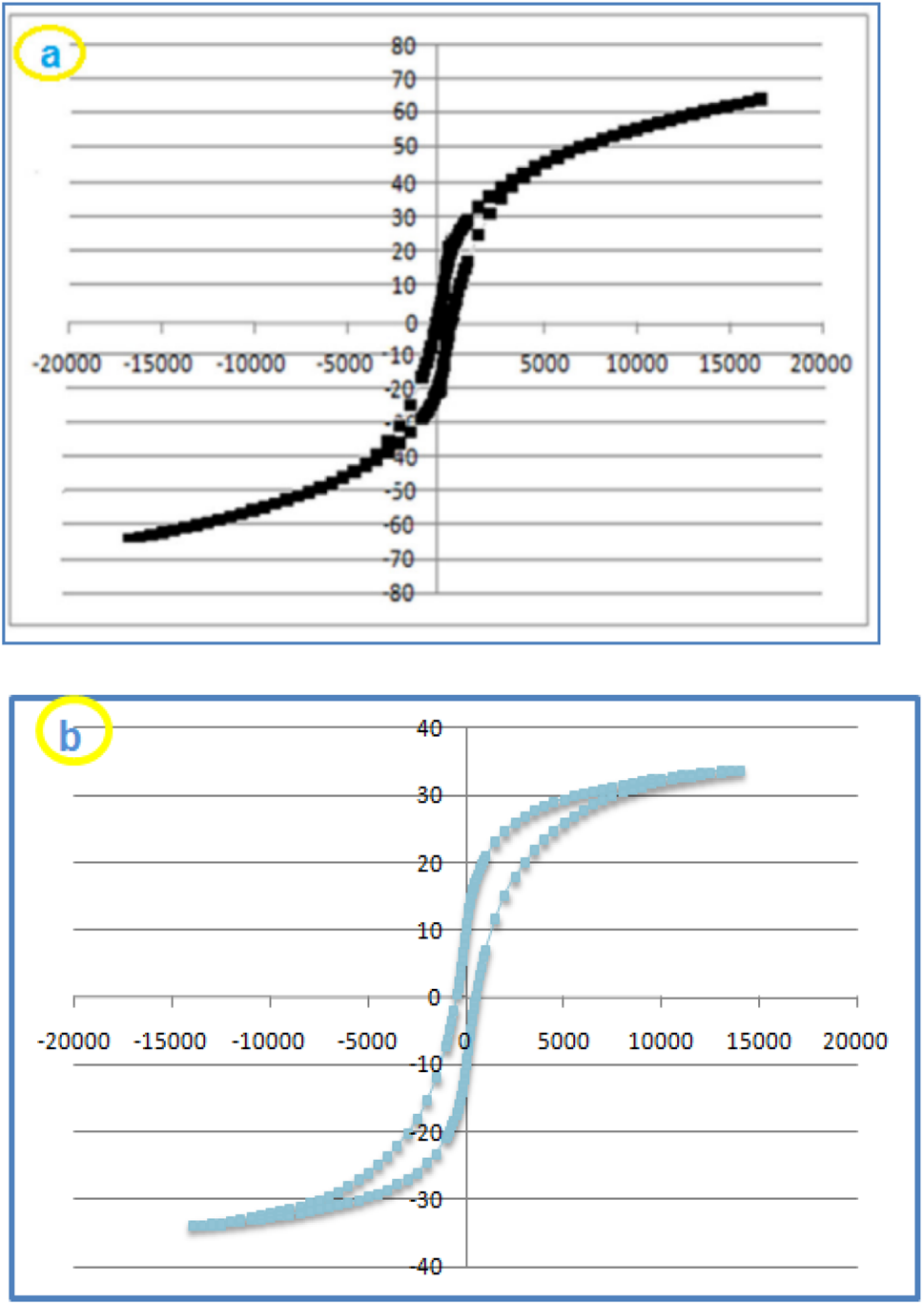
Magnetization curves of CoFe_2_O_4_ (a) and CoFe_2_O_4_@HPECG/Pr-SO_3_H·Cu(ii) at ambient temperature (b).

Vibrating-sample magnetometry (VSM) is an efficient tool to characterize the magnetic feature of CoFe_2_O_4_ (a) and CoFe_2_O_4_@HPECG/Pr-SO_3_H·Cu(ii) (b). The saturation magnetization value (*M*_s_) of CoFe_2_O_4_ and CoFe_2_O_4_@HPECG/Pr-SO_3_H were about 65.53 and 34.02 emu g^−1^, respectively (Fig. 3). As a result of this analysis, the *M*_s_ value for CoFe_2_O_4_ is higher than catalyst which is due to the existence of HPECG/Pr-SO_3_H and Cu supported on nanoparticles. Although, this magnetic property of CoFe_2_O_4_@HPECG/Pr-SO_3_H·Cu(ii) is still enough to ensure that the nanocatalyst quickly recovers from the reaction mixture using an external magnet.

#### 3.1.4. X-ray diffraction

**Fig. 4:**
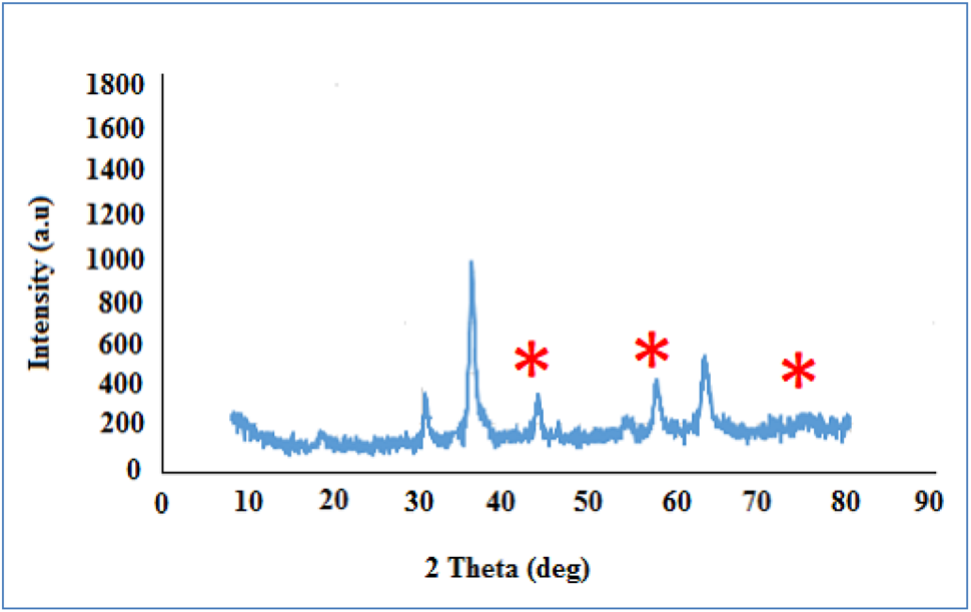
X-ray diffraction (XRD) pattern of CoFe_2_O_4_@HPECG/Pr-SO_3_H·Cu(ii).

The X-ray diffraction (XRD) pattern of the structure of the CoFe_2_O_4_@HPECG/Pr-SO_3_H·Cu(ii) composite is displayed in Fig. 4. According to the existing patterns, it appears that the amorphous pectin obtained from lemon peel has only one peak at an angle of approximately 2*θ* ≈ 18.12 and 32.12°, and the peaks with low intensity confirm the amorphous nature of the substrate. The diffraction peaks observed for Bragg's reflections from planes (2 2 0), (3 1 1), (4 0 0), (4 2 2), (3 3 3), and (4 4 0) match the standard spinel structure of CoFe_2_O_4_ (JCPDS card No. 22-1086).^39^ Additionally, the peak appearing at angles 2*θ* ≈ 43.9, 57.4, and 74.8 is associated with the diffraction of the (1 1 1), (2 0 0), and (2 2 0) planes of metallic copper with a face-centered cubic structure (marked as asterisk).^40^ Based on the Debye–Scherrer equation, the average size of these CoFe_2_O_4_@HPECG/Pr-SO_3_H·Cu(ii) particles is calculated to be nearly 21 nm.

The accurate value of Cu loaded on modified CoFe_2_O_4_@HPECG/Pr-SO_3_H, ICP analysis was performed and it was measured to be 0.33 mmol g^−1^. Also, the exact amount of the sulfur for CoFe_2_O_4_@HPECG/Pr-SO_3_H·Cu(ii) was found to be 0.84 mmol g^−1^ by the aid of the CHNS method.^41^

#### 3.1.5. FT-IR spectroscopy

**Fig. 5:**
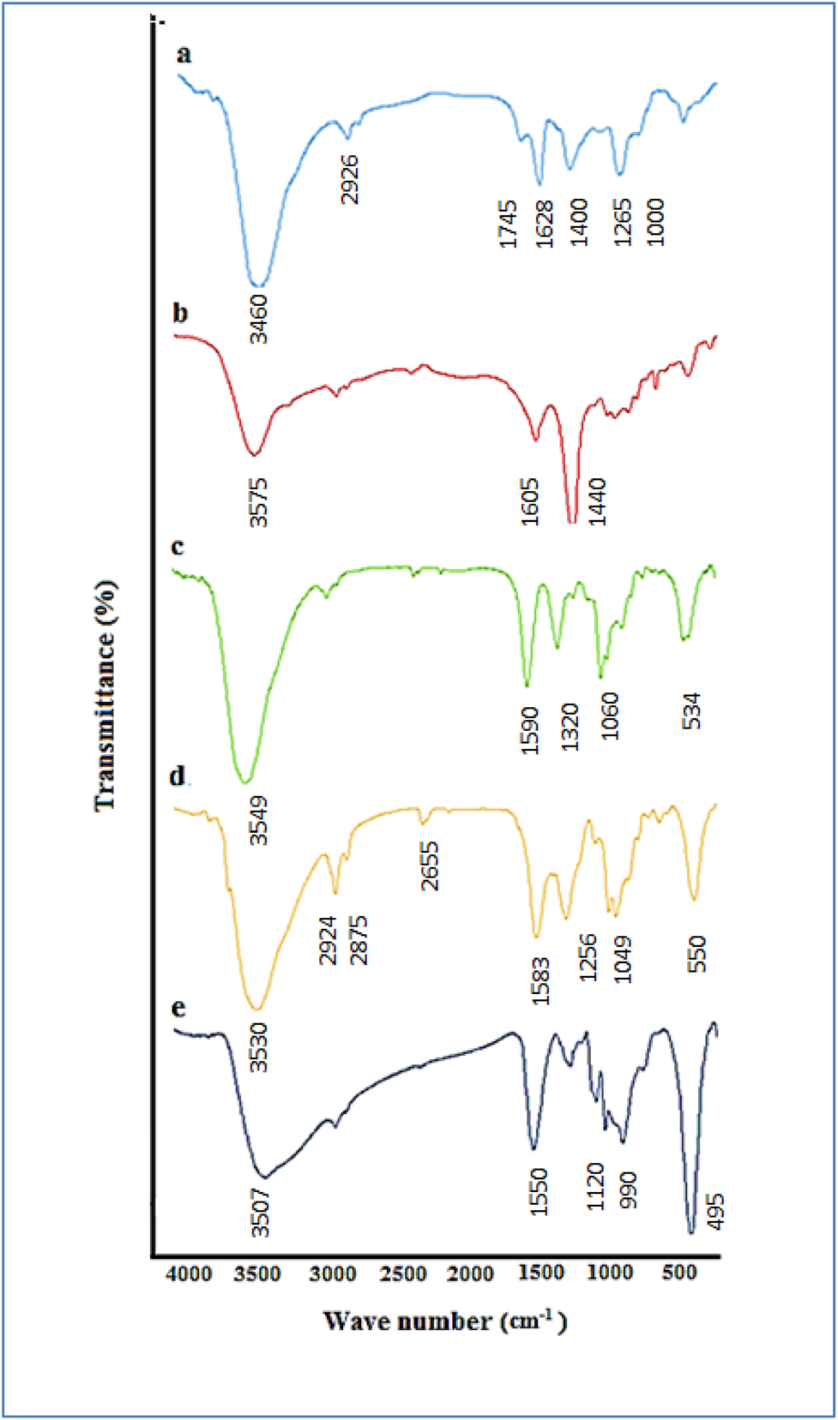
FT-IR spectra of PEC (a), HPECG (b), CoFe_2_O_4_@HPECG (c), CoFe_2_O_4_@HPECG/Pr-SO_3_H (d), and CoFe_2_O_4_@HPECG/Pr-SO_3_H·Cu(ii) (e).

FT-IR techniques, as demonstrated in Fig. 5, are able to describe and verify the preparation of the catalyst. In the comparative FT-IR spectra of PEC (a), HPECG (b), CoFe_2_O_4_@HPECG (c), CoFe_2_O_4_@HPECG/Pr-SO_3_H (d), and CoFe_2_O_4_@HPECG/Pr-SO_3_H·Cu(ii) (e), the broad peak observed in the 3460 cm^−1^ region of the FT-IR spectra is attributed to the stretching vibrations of the O–H groups, and the stretching vibration of C–H is observed in the 2926 cm^−1^ region. The peak in the 1745 cm^−1^ region is related to the vibration of the carbonyl groups of esters, and the peak corresponding to the carboxylic acid groups is seen in the 1628 cm^−1^ region.^39^ Peaks related to the stretching vibrations of the C–O and C=C groups in the pectin molecule are observed in the 1000–1265 cm^−1^ region (Fig. 5a). The hydrolysis of ester groups into acidic groups results in the disappearance of the peak at 1745 cm^−1^ (Fig. 5b).^42^ Also, a shift of the carbonyl groups to lower frequencies is observed, which is attributed to a strong interaction between calcium ions and pectin molecules for the formation of a hydrogel.

The peaks at 448 cm^−1^ and 534 cm^−1^ represent the stretching vibrations of Fe–O bonds in the tetrahedral and octahedral sites of CoFe_2_O_4_, respectively (Fig. 5c).

Additionally, new broad bonds are observed at 1049, 1256, 2655, and 2924 cm^−1^. These can be attributed to MPTMS groups attached to the surface of CoFe_2_O_4_@HPECG (Fig. 5d).

#### 3.1.6. Thermal gravimetric analysis

**Fig. 6:**
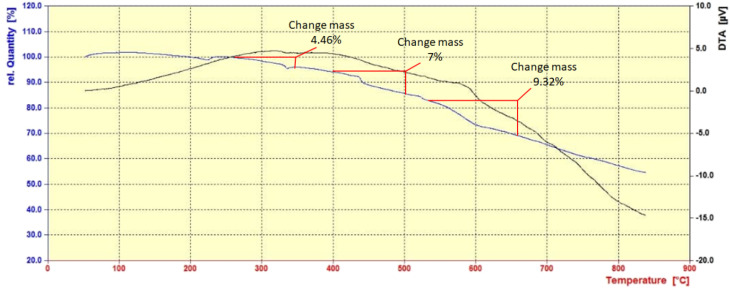
TGA and DTA curve of CoFe_2_O_4_@HPECG/Pr-SO_3_H·Cu(ii).

The stability of the synthesized nanocatalyst CoFe_2_O_4_@HPECG/Pr-SO_3_H·Cu(ii) was measured by TGA and DTG in the temperature range of 25–850 °C. In the TGA curve (Fig. 6), the nanocatalyst CoFe_2_O_4_@HPECG/Pr-SO_3_H·Cu(ii) shows its first weight loss around 46.4%, which corresponds to the loss of solvent molecules adsorbed on the pectin substrate. The next weight loss, around 7%, is related to the decomposition of organic groups on the CoFe_2_O_4_@HPECG/Pr-SO_3_H substrate. Although the bonding of organic groups reduces its thermal stability, the nanocatalyst remains stable up to 220 °C. Molecular decomposition of this catalyst begins at 220 °C, making it a suitable catalyst for reduction reactions that occur at lower temperatures.

#### 3.1.7. N_2_ adsorption–desorption isotherms studies

The surface area and pore size of the particles were analyzed using BET adsorption isotherm and Barrett–Joyner–Halenda (BJH) techniques.

**Fig. 7:**
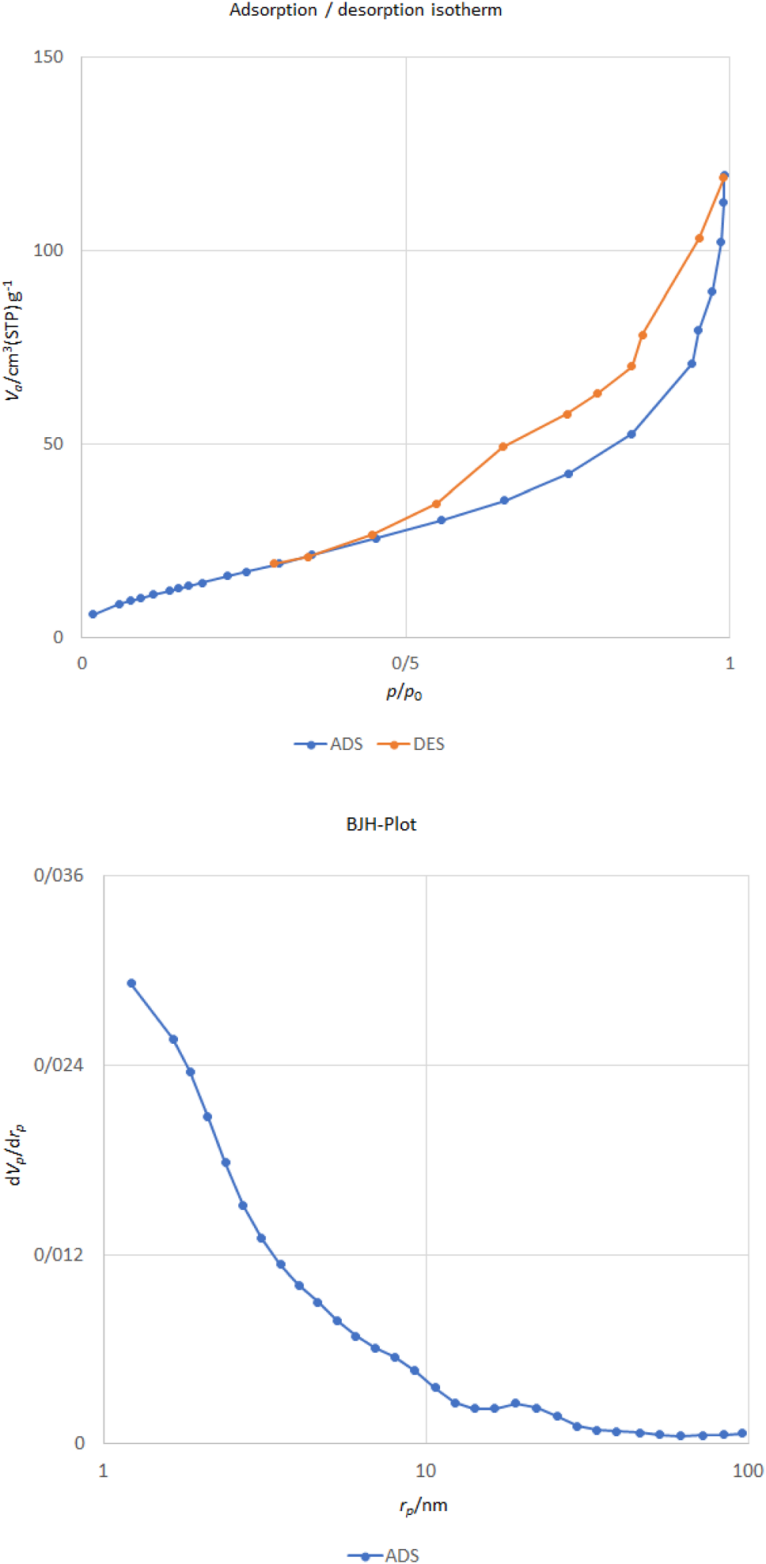
Nitrogen adsorption–desorption isotherms and BJH pore size distributions of CoFe_2_O_4_@HPECG/Pr-SO_3_H·Cu(ii).

The N_2_ sorption isotherm of the CoFe_2_O_4_@HPECG/Pr-SO_3_H·Cu(ii) catalyst is presented in Fig. 7. The sample demonstrate isotherm type IV with a type H_3_ hysteresis loop according to the IUPAC classification of adsorption isotherms. As indicated by the BET analysis of this catalyst, it shows an effective surface area of 60.32 m^2^ g^−1^, which is reduced compared to the surface area of pectin ^43^. Additionally, calculations based on the BJH plot from the nitrogen adsorption–desorption branch showed that the pore volume and pore size for the CoFe_2_O_4_@HPECG/Pr-SO_3_H·Cu(ii) catalyst is 0.18 cm^3^ g^−1^ and 11.67 nm, respectively. The agglomeration of nanoparticles may be the reason of decline in the surface area, also the decrease in pore volume and pore size is due to the fact that the HPECG/Pr-SO_3_H and Cu are loaded onto the CoFe_2_O_4_ surface.

### 3.2. Catalytic activity

Aromatic amines play a crucial role as key intermediates in the synthesis of dyes, antioxidants, pharmaceuticals, and photographic materials. One of the most common and effective methods for preparing amines is the reduction of nitroarenes. Extensive research has been conducted on the process of reducing nitroarenes to aromatic amines, and a fundamental problem observed in most employed methods is the lack of appropriate selectivity among the reduction products. Sodium borohydride is one of the most commonly used reducing agents for nitro compounds, typically used in the presence of a metal catalyst.

In the first stage of the catalytic activity of CoFe_2_O_4_@HPECG/Pr-SO_3_H·Cu(ii) in the reaction of nitroarenes to amines, the effect of various amounts of sodium borohydride, different temperatures, and different solvents were studied. The results of this study are presented in Table 1.

**Table 1:** Optimization experiments for reduction of nitrobenzene using CoFe_2_O_4_@HPECG/Pr-SO_3_H·Cu(ii) nanocatalyst

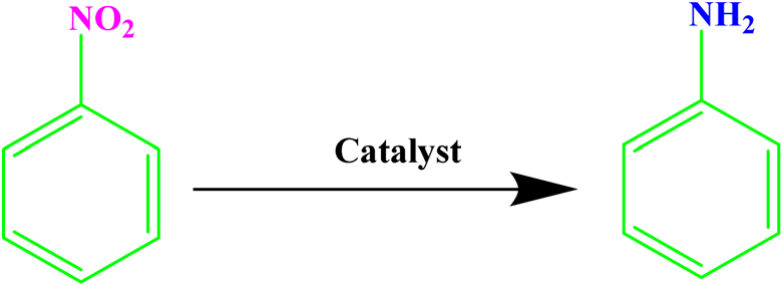					
Entry	Catalysts among (mg)	Reducing agent	Condition	Time (min)	Yield^a^ (%)
1	25	NaBH_4_ (0.15 mmol)	CH_3_CN/r.t.	100	—
2	25	NaBH_4_ (0.15 mmol)	*n*-Hexane/r.t.	120	—
3	25	NaBH_4_ (0.15 mmol)	Toluene/r.t.	80	—
4	25	NaBH_4_ (0.15 mmol)	CH_2_Cl_2_/r.t.	80	—
5	25	NaBH_4_ (0.15 mmol)	EtOAc/r.t.	85	40
6	25	NaBH_4_ (0.15 mmol)	H_2_O/r.t.	5	97
7	25	NaBH_4_ (0.15 mmol)	EtOH/r.t.	15	77
8	—	NaBH_4_ (0.15 mmol)	H_2_O/r.t.	100	—
9	25	NaBH_4_ (0.1 mmol)	H_2_O/r.t.	10	97
10	25	NaBH_4_ (0.08 mmol)	H_2_O/r.t.	30	85
11	25	NaBH_4_ (0.06 mmol)	H_2_O/r.t.	45	79
12	25	NaBH_4_ (0.05 mmol)	H_2_O/r.t.	45	68
13	25	—	H_2_O/r.t.	100	—
14	20	NaBH_4_ (0.15 mmol)	H_2_O/r.t.	45	89
15	30	NaBH_4_ (0.15 mmol)	H_2_O/r.t.	120	35
16	CoFe_2_O_4_ (25)	NaBH_4_ (0.15 mmol)	H_2_O/r.t.	80	25
17	CoFe_2_O_4_@HPECG/Pr-SO_3_H (25)	NaBH_4_ (0.15 mmol)	H_2_O/r.t.	60	30
18	HPECG (25)	NaBH_4_ (0.15 mmol)	H_2_O/r.t.	120	—
19	CuCl_2_·2H_2_O (25)	NaBH_4_ (0.15 mmol)	H_2_O/r.t.	65	33

a Isolated yield.

The best result is in the reduction of nitroarenes to amines using the CoFe_2_O_4_@HPECG/Pr-SO_3_H·Cu(ii) catalyst was achieved with 0.15 mmol of sodium borohydride in water as the solvent, leading to a high-efficiency reaction in the shortest time. This finding was confirmed using TLC. The reaction of nitroarenes with various amounts of sodium borohydride was studied. It was observed that in the absence of sodium borohydride, the reaction did not progress, but with an increase in the amount of sodium borohydride, a significant and efficient result was achieved in a shorter time.

To assess the catalytic effect, the reaction was conducted in the absence of the catalyst in water solvent with 0.1 mmol of sodium borohydride. After 100 min, the reaction yielded no significant result. The findings indicate that the reaction proceeds with higher efficiency and speed in polar solvents compared to non-polar solvents. Water was chosen as the suitable solvent for this reaction, suggesting that water plays a crucial role not only as a solvent but also as a promoter in this reaction.

The reaction was carried out with CoFe_2_O_4_, CoFe_2_O_4_@HPECG/Pr-SO_3_H and HPECG as a catalyst, but it did not result in any progress even after an extended period. Additionally, when the CoFe_2_O_4_@HPECG/Pr-SO_3_H·Cu(ii) nanocatalyst was replaced with CuCl_2_·2H_2_O, the resulted product was produced in a lower yield (33%).

After optimizing the reaction conditions, various nitroarenes were examined in this method to expand its scope. In the presence of the CoFe_2_O_4_@HPECG/Pr-SO_3_H·Cu(ii) catalyst, the corresponding derivatives were synthesized with high yields (as shown in Table 2).

**Table 2:** Reduction of nitro compounds with NaBH_4_ in the presence of CoFe_2_O_4_@HPECG/Pr-SO_3_H·Cu(ii) nanocatalyst

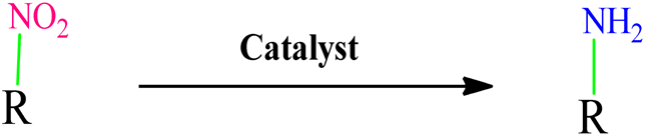					
Entry	Nitro compound	Product	Time (min)	Yield^a^ (%)	m.p. (°C)
1	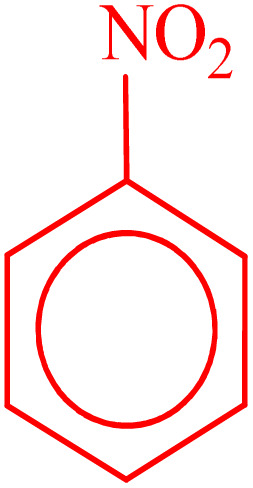	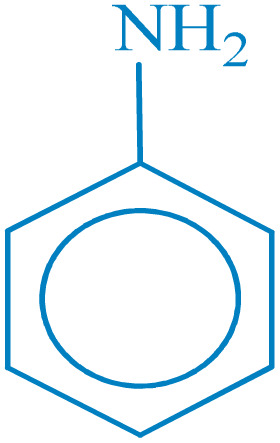	5	97	Oil
2	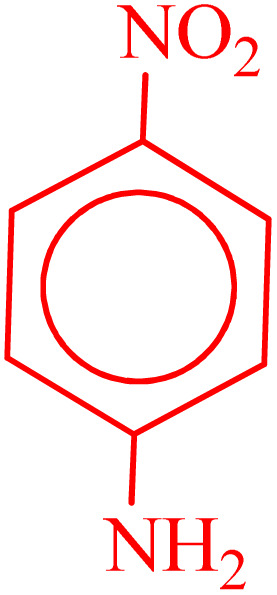	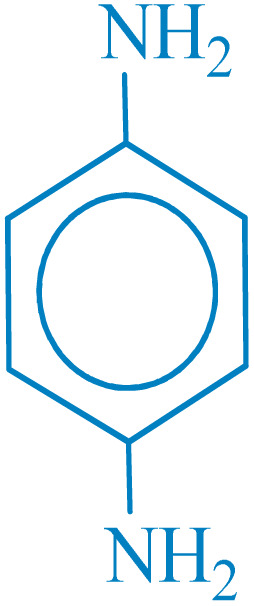	5	95	141
3	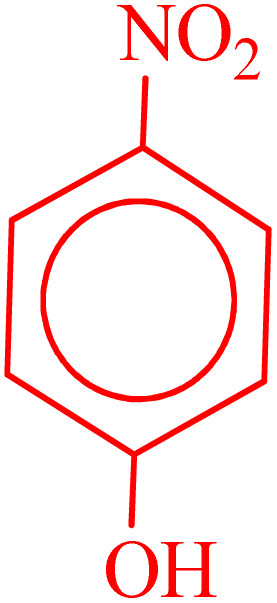	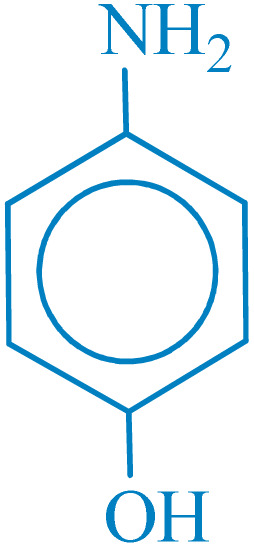	9	93	190
4	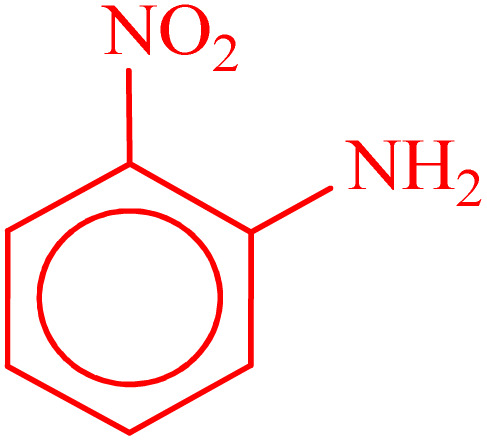	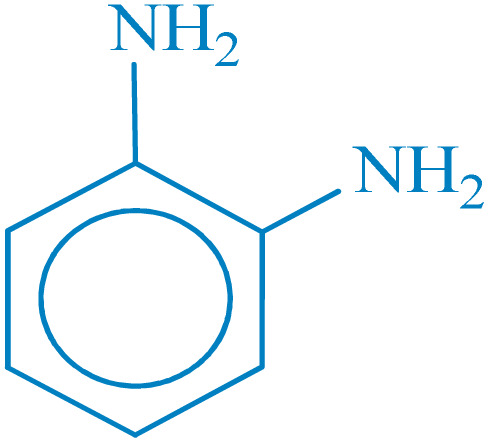	8	90	105
5	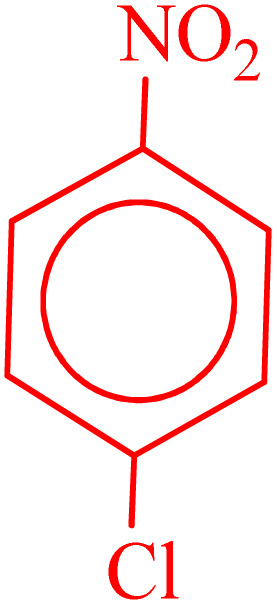	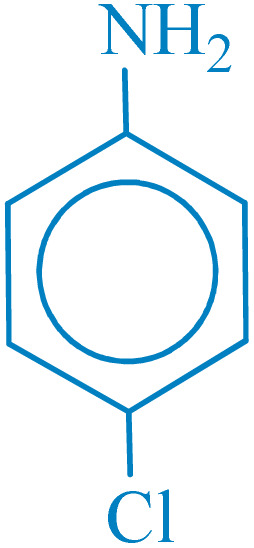	13	91	Oil
6	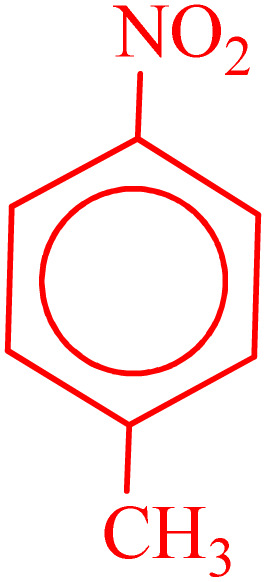	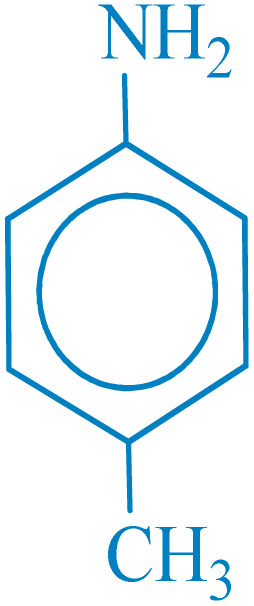	8	94	Oil
7	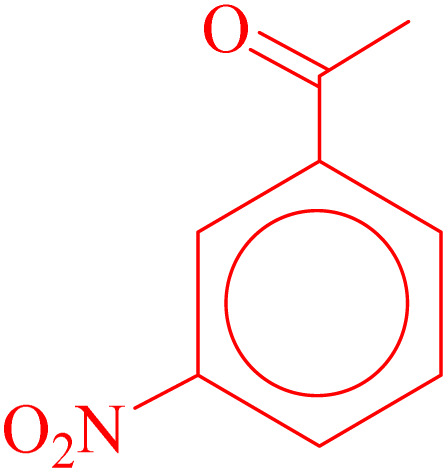	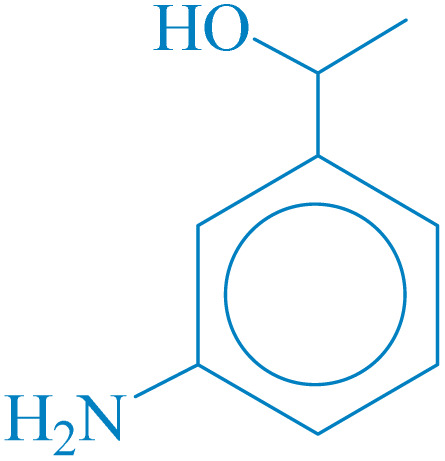	7	92	Oil
8	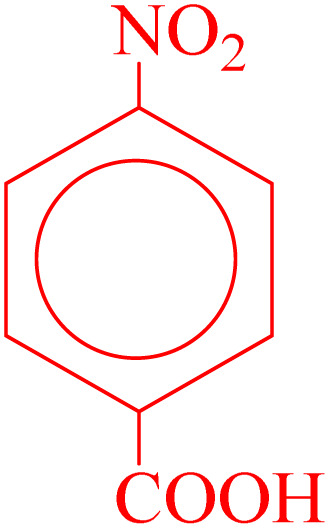	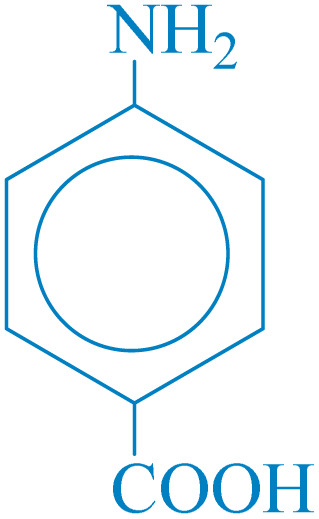	10	91	181
9	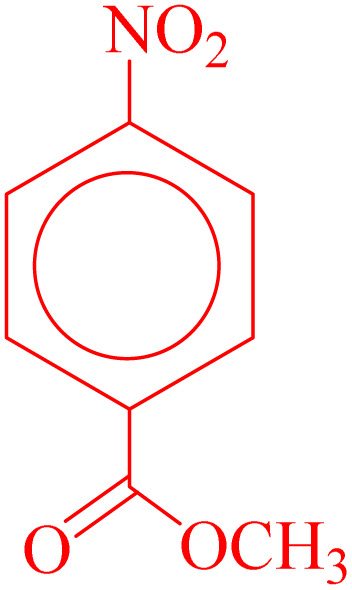	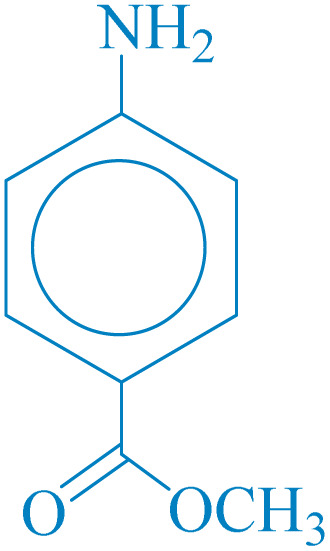	15	93	105

a Isolated yields.

In this method, various types of nitroarenes with electron-donating and electron-withdrawing groups were efficiently and rapidly converted into their corresponding aromatic amines. The results indicate that electron-donating substituents, compared to electron-withdrawing ones, require more time for the reduction to the respective amines.

**Scheme 2:**
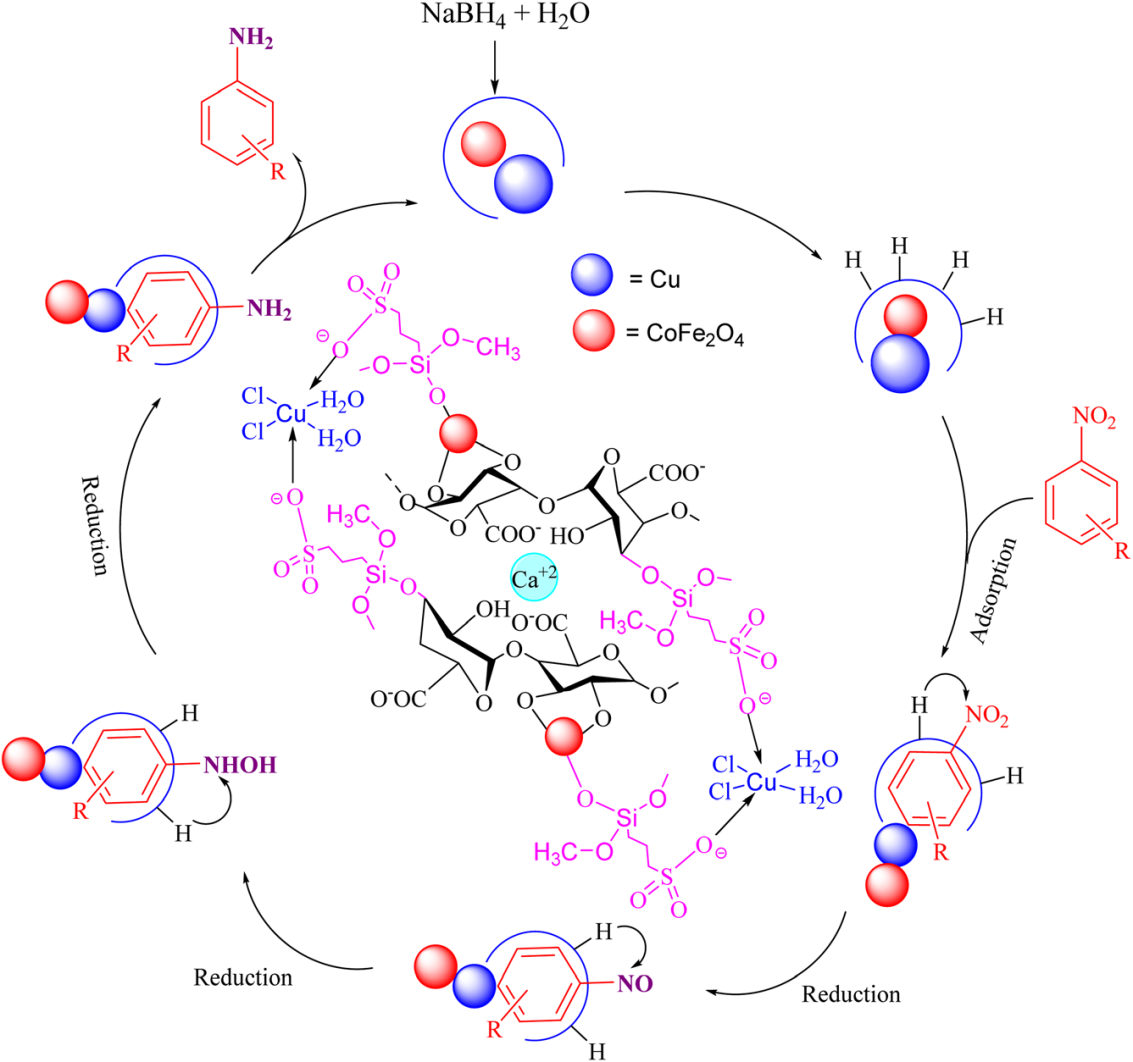
A plausible reaction mechanism for reduction of nitrobenzene.

A plausible mechanism for the catalytic activity of nano CoFe_2_O_4_@HPECG/Pr-SO_3_H·Cu(ii) is depicted in Scheme 2. As can be seen, the magnetic CoFe_2_O_4_ nanoparticles are deposited on the surface of the pectin hydrogel, allowing for easy separation of the magnetic nanoparticles within the pectin matrix. On the other hand, Cu(ii) ions bind with the sulfonic acid groups in the nanocomposite, creating active sites in the CoFe_2_O_4_@HPECG/Pr-SO_3_H nanocomposite *via* Cu(ii) for the reaction to proceed. The nanocatalyst facilitates the reduction of nitro groups, involving four distinct steps in the nitro reduction process. Initially, hydrogen absorption takes place, followed by adsorption on the metal surfaces. In the third stage, there is an electron transfer through metal surfaces from BH_4_^−^ to aromatic nitro compounds. Subsequently, aromatic amino compounds desorb from the catalyst surface. During this process, B–H bond cleavage occurs on the surface of CoFe_2_O_4_@HPECG/Pr-SO_3_H·Cu(ii) nanocatalyst.

The activity of CoFe_2_O_4_@HPECG/Pr-SO_3_H·Cu(ii) nanocatalyst in the synthesis of tetrazoles was also investigated. It should be noted that the reaction between sodium azide (1.2 mmol) and benzene nitrile (1 mmol) using CoFe_2_O_4_@HPECG/Pr-SO_3_H·Cu(ii) was selected as a sample reaction.

To optimize the reaction conditions, the influence of catalyst amount, temperature, and solvent on the sample's reaction was examined. As can be seen in Table 3, the effect of the different solvents such as EtOH, H_2_O, DMSO, Toluene, DMF was studied. The use of a protic polar solvent will raise the reaction rate and it was discovered that H_2_O serves as an appropriate solvent for this reaction (Table 3, entries 1–5). To determine the optimal reaction temperature, this procedure was examined at various temperatures, and 70 °C being the most effective (Table 3, entries 5–8). Also, it was observed that the reaction at the room temperature produced the highest time and the lowest yield (Table 3, entry 12). Furthermore, the reaction's output was maintained at temperatures exceeding 70 °C (Table 3, entry 5). Then, the effect of the catalyst amount on the reaction yield was investigated. The results displayed that the use of 40 mg of CoFe_2_O_4_@HPECG/Pr-SO_3_H·Cu(ii) nanocatalyst is sufficient to complete the reaction in 20 min (Table 3, entries 8–10). Meanwhile, the reaction did not proceed at all without the catalyst, even after 100 min (Table 3, entry 11). This result confirms that optimum conditions in the synthesis of tetrazoles as a sole product in high yield was in the presence of H_2_O solvent at 70 °C in the 40 mg of CoFe_2_O_4_@HPECG/Pr-SO_3_H·Cu(ii) nanocatalyst. Also shows that the catalytic activity of CoFe_2_O_4_, CoFe_2_O_4_@HPECG/Pr-SO_3_H and HPECG for the synthesis of 1*H*-tetrazole was not note-worthy, even after a long period, there was not result (Table 3, entries 13–15). Furthermore, the product was produced in low yield in the presence of CuCl_2_·2H_2_O (Table 3, entry 16).

**Table 3:** Optimization experiments for synthesis of 1*H*-tetrazole using CoFe_2_O_4_@HPECG/Pr-SO_3_H·Cu(ii) nanocatalyst^a^

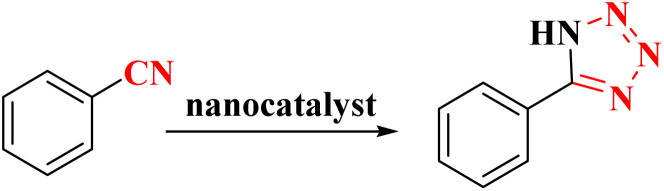					
Entry	Solvent	Catalyst (mg)	Temperature (°C)	Time (min)	Yield^b^ (%)
1	DMF	40	100	60	87
2	EtOH	40	80	43	82
3	Toluene	40	100	115	76
4	DMSO	40	100	95	85
5	H_2_O	40	80	15	95
6	H_2_O	40	60	25	92
7	H_2_O	40	40	50	61
8	H_2_O	40	70	20	98
9	H_2_O	50	70	15	75
10	H_2_O	20	70	45	62
11	H_2_O	—	70	100	—
12	H_2_O	40	Room temperature	120	40
13	H_2_O	CoFe_2_O_4_ (40)	70	70	20
14	H_2_O	CoFe_2_O_4_@HPECG/Pr-SO_3_H (40)	70	80	35
15	H_2_O	HPECG (40)	70	100	—
16	H_2_O	CuCl_2_·2H_2_O (40)	70	60	40

a Reaction conditions: NaN_3_ (1.2 mmol), Nitrile (1 mmol), CoFe_2_O_4_@HPECG/Pr-SO_3_H·Cu(ii) (40 mg), and solvent (3 mL).

b Isolated yield.

After obtaining the appropriate optimal conditions, several tetrazole derivatives were synthesized in the presence of the CoFe_2_O_4_@HPECG/Pr-SO_3_H·Cu(ii) (Table 4). The products were obtained with short reaction time and high efficiency.

**Table 4:** Synthesis of 5-substituted 1*H*-tetrazoles derivatives in the presence of CoFe_2_O_4_@HPECG/Pr-SO_3_H·Cu(ii) nanocatalyst

Entry	Substrate	Product	Time (min)	Yield^a^ (%)	m.p. (°C)
1	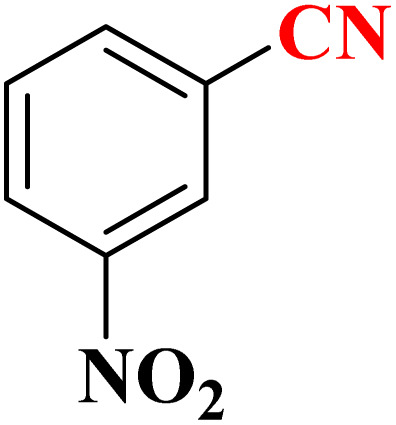	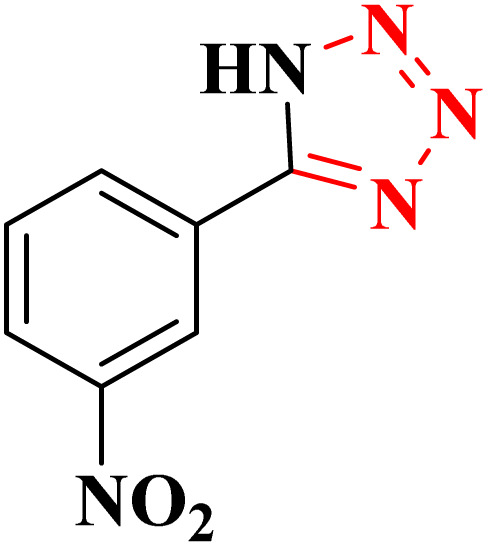	22 h	65	149–152 (ref. 36)
2	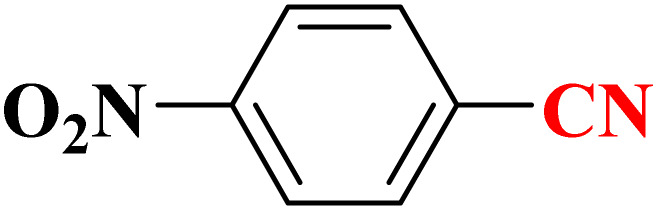	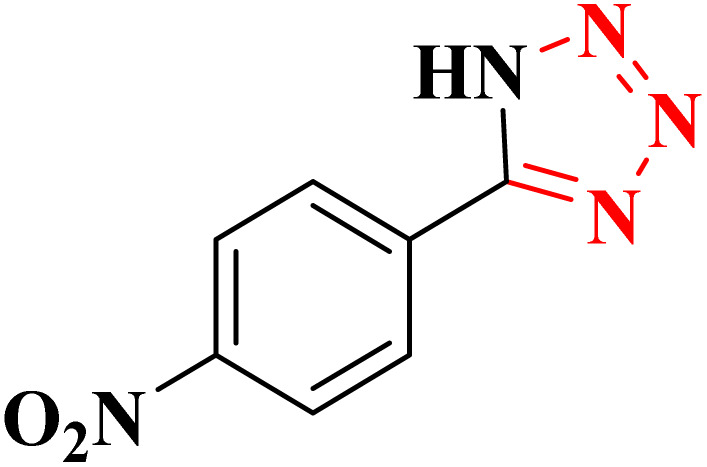	22 h	75	218–220 (ref. 36)
3	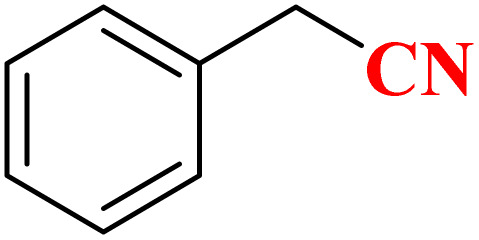	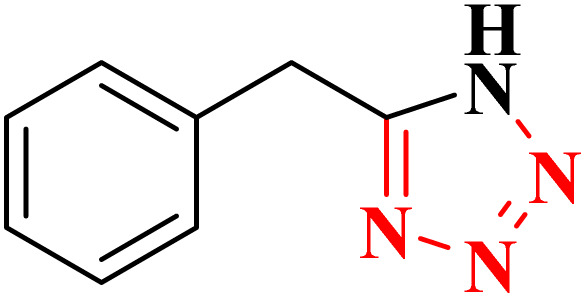	33	55	123–126 (ref. 36)
4	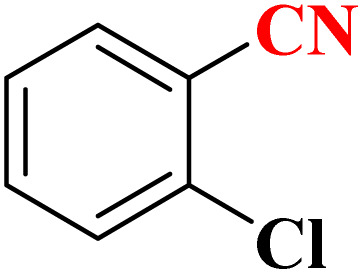	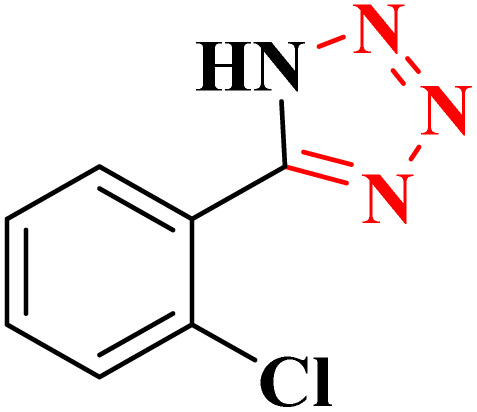	22	52	179–180 (ref. 36)
5	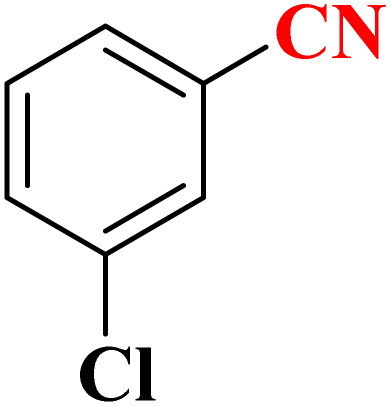	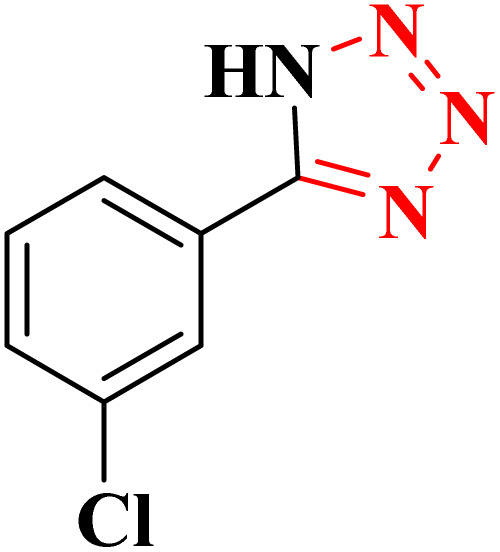	22	56	132–134 (ref. 36)
6	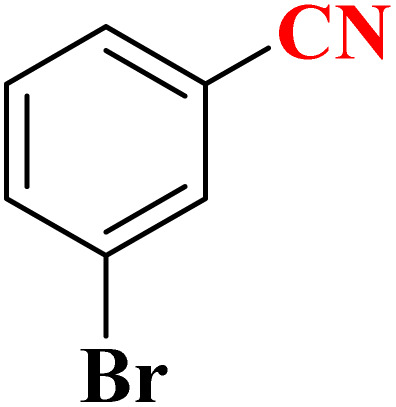	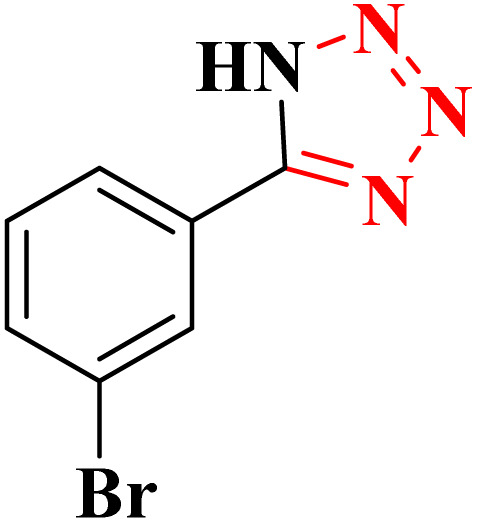	25	68	182–185
7	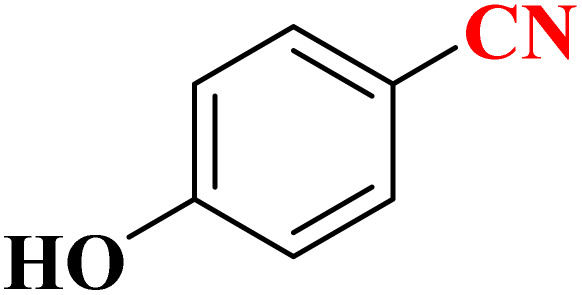	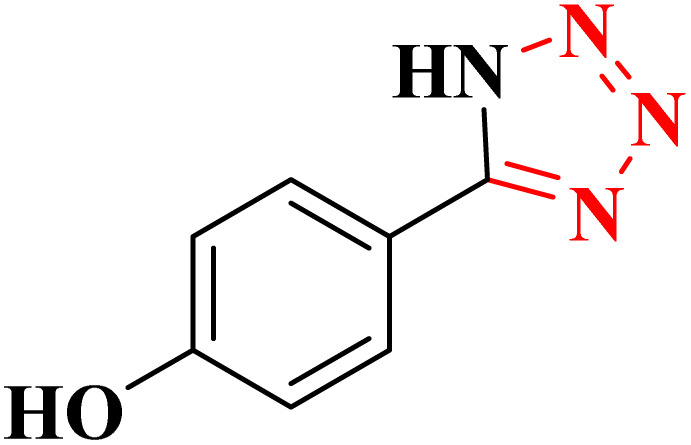	24 h	75	233–235
8	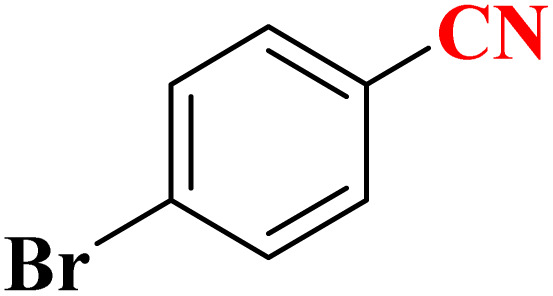	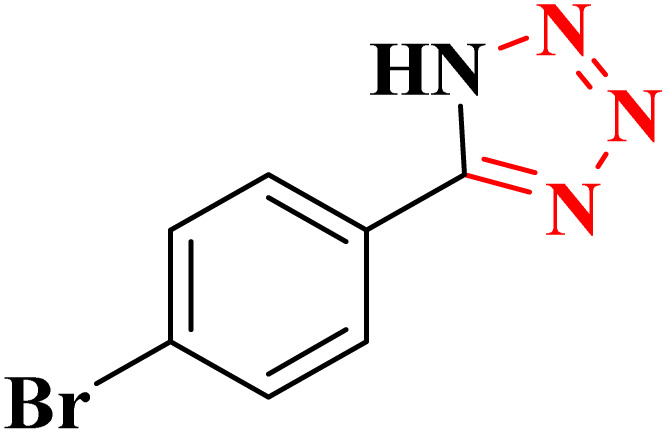	20	58	263–266 (ref. 36)
9	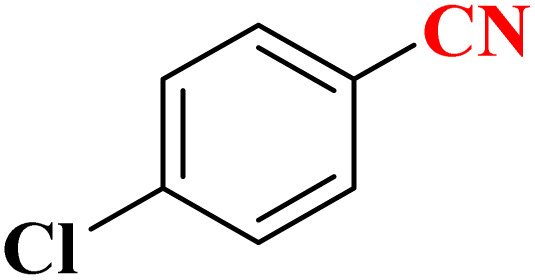	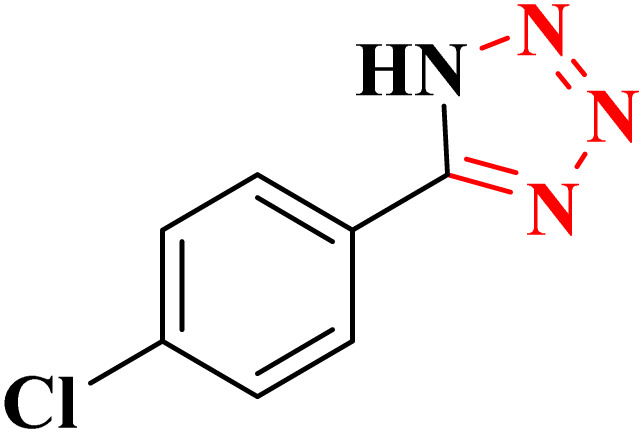	25	80	261–264 (ref. 36)
10	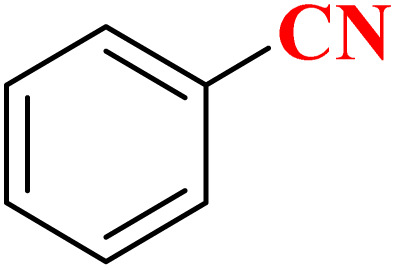	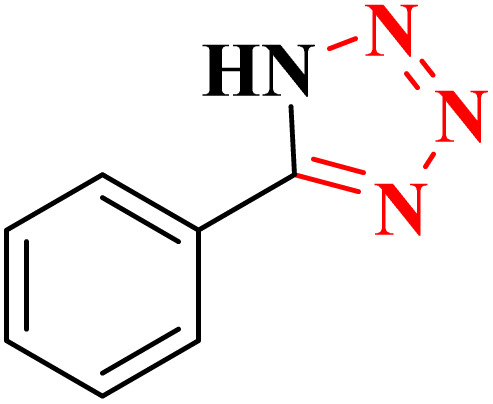	20	98	214–216 (ref. 36)

a Isolated yields.

**Scheme 3:**
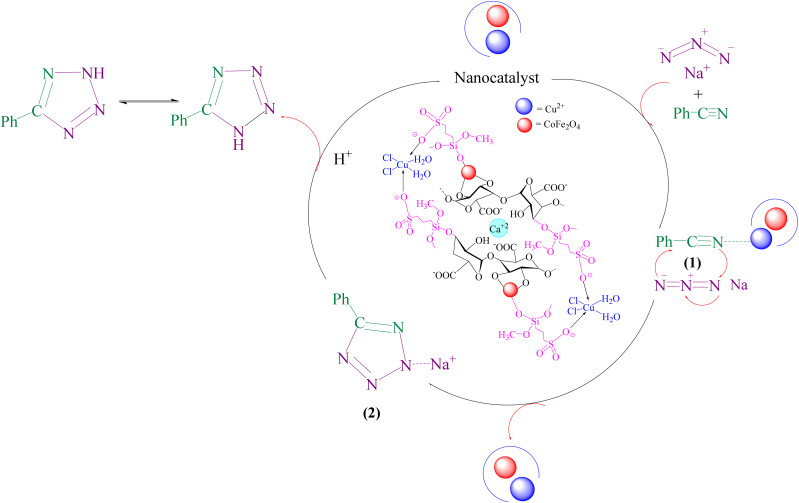
A plausible reaction mechanism for synthesis of tetrazole.

A suggested mechanism for examining the catalytic reaction is illustrated in Scheme 3. In this mechanism, the Lewis acidic copper serves as the active site for coordinating the nitrile molecule. The [3 + 2] cycloaddition nanocatalyzed tetrazole synthesis was initiated with interaction of nitrile group with Cu^2+^ of nanocatalyst and forms an intermediate which accelerates [3 + 2] cycloaddition step. Certainly, nanocatalyst activates nitrile groups *via* coordination to nitrogen and/or triple bond which enhances electrophilic character of cyanide group (intermediate 1). Thereafter, sodium azide reacts with this complex and produces second intermediate (2). Finally, 1,3-*H*-shift produce tetrazole product with acidic work up.

### 3.3. Comparison of the catalyst

The comparison of the CoFe_2_O_4_@HPECG/Pr-SO_3_H·Cu(ii) nanocatalyst with other reported catalysts for the reduction of nitroarenes to amines (Table 5, entries 1–5), and synthesis of 1*H*-tetrazoles (Table 6, entries 1–5) is presented. Recently, the use of Ag nanoparticles deposition on magnetic α-Fe_2_O_3_ nanocrystals surface by the reduction of AgNO_3_ with NaBH_4_ (Table 5, entry 2), zinc phthalocyanine with PEG-400 (Table 5, entry 3), Ag nanoparticles incorporated β-cyclodextrin conjugated magnetic hydroxyapatite (Table 5, entry 4), immobilization of bimetallic Fe–Cu on microcrystalline cellulose (Table 5, entry 5) as catalyst have been reported for reduction of nitroarenes to amines. These procedures require high-temperature, longer reaction times, with high amounts of catalyst.

**Table 5:** Comparison of catalytic activity of CoFe_2_O_4_@HPECG/Pr-SO_3_H·Cu(ii) for the reduction of nitroarenes with the some recently reported procedures

Entry	Catalyst (mg)	Time (min)	Reaction conditions	Yield^a^ (%)	Ref.
1	CoFe_2_O_4_@HPECG/Pr-SO_3_H·Cu(ii) (25)	5	NaBH_4_, H_2_O, r.t.	97	This work
2	Fe_2_O_3_/Ag (1)	30	NaBH_4_, H_2_O, 100 °C	99	44
3	Zinc phthalocyanine PEG-400 (1)	8	NaBH_4_, EtOH, 100 °C	99	42
4	γ-Fe_2_O_3_@HAp-CD·Ag (5)	30	NaBH_4_, H_2_O, 80 °C	98	45
5	Fe–Cu@MCC (3)	8	NaBH_4_, H_2_O, 70 °C	93	46

a Isolated yield of product.

**Table 6:** Comparison of catalytic activity of CoFe_2_O_4_@HPECG/Pr-SO_3_H·Cu(ii) for the synthesis of 1*H*-tetrazole with the some recently reported procedures

Entry	Catalyst (mg)	Time (min)	Reaction conditions	Yield^a^ (%)	Ref.
1	CoFe_2_O_4_@HPECG/Pr-SO_3_H·Cu(ii) (40)	20	H_2_O, 70 °C	98	This work
2	CuO-NrGO (10 mg)	300	DMF, 140 °C	91	47
3	CS@Tet-IL-Cu(ii) (50 mg)	20	DMF, 120 °C	83	48
4	CoFe_2_O_4_/Ser/Cu (100 mg)	4	EtOH/H_2_O, 75 °C	65	49
5	BNPs@Cur-Ni (40 mg)	75	PEG, 120 °C	97	50

a Isolated yield.

In addition, the use of copper oxide nitrogen-doped reduced graphene oxide (CuO-NrGO) nanoparticles (Table 6, entry 2), 1-phenyl-1*H*-tetrazole-5-thiol ionic liquid Cu(ii) complex has been supported on chitosan using (3-chloropropyl)trimethoxysilane (Table 6, entry 3), Copper catalysts supported on CoFe_2_O_4_ and MCM-41 with serine ligands (Table 6, entry 4), the extracted curcumin for surface modification boehmite nanoparticles (BNPs) to anchor nickel ions (Table 6, entry 5) as catalyst have been reported for synthesis of 1*H*-tetrazoles. These procedures require toxic solvents, higher reaction times, lower product yields and high amounts of catalyst.

As can be seen, this catalyst CoFe_2_O_4_@HPECG/Pr-SO_3_H·Cu(ii) has attractive features such as suitable reaction times, high yields, and green solvent.

### 3.4. Reusability test of magnetic catalyst

**Fig. 8:**
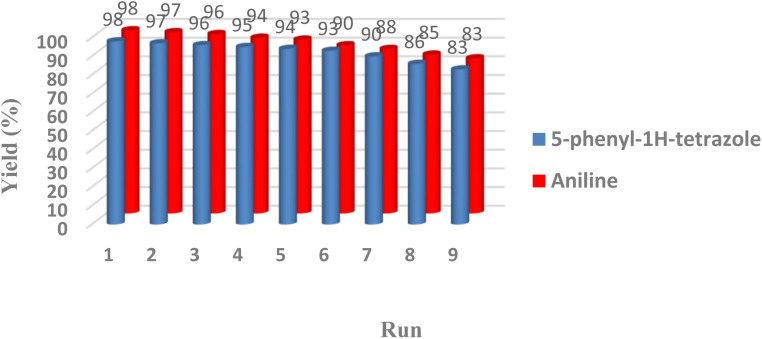
Recyclability of CoFe_2_O_4_@HPECG/Pr-SO_3_H·Cu(ii) in the synthesis of aniline and 5-phenyl-1*H*-tetrazole.

Reusability is one of the most important properties of the applied nanomagnetic catalysts was also checked in the one-pot conversion of nitrile to tetrazole with benzonitrile (1 mmol), catalyst (40 mg), NaN_3_ (1.2 mmol), and reduction of nitroarenes to amines with nitrobenzene (1 mmol), sodium borohydride (0.15 mmol), catalyst (25 mg) in an aqueous medium. After completion of the reaction, CoFe_2_O_4_@HPECG/Pr-SO_3_H·Cu(ii) nanocomposite was separated magnetically from the reaction mixture and used in the next cycles under optimum conditions. It can be seen in Fig. 8, a slight decrease in the activity of the catalyst was seen after utilizing nine cycles that exhibited good stability of CoFe_2_O_4_@HPECG/Pr-SO_3_H·Cu(ii) nanocomposite under the reaction conditions. The yield of the transformations diminished in nine runs, whereas the selectivity of the catalyst remains constant.

### 3.5. Large-scale synthesis and recycling experiments for model reaction

**Scheme 4:**
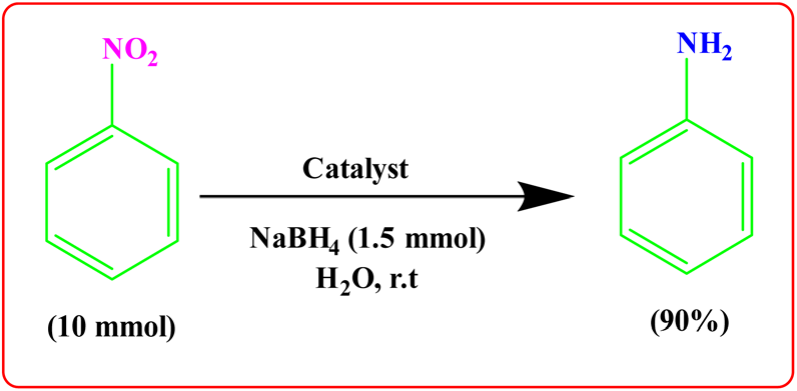
Large-scale synthesis of aniline.

**Scheme 5:**
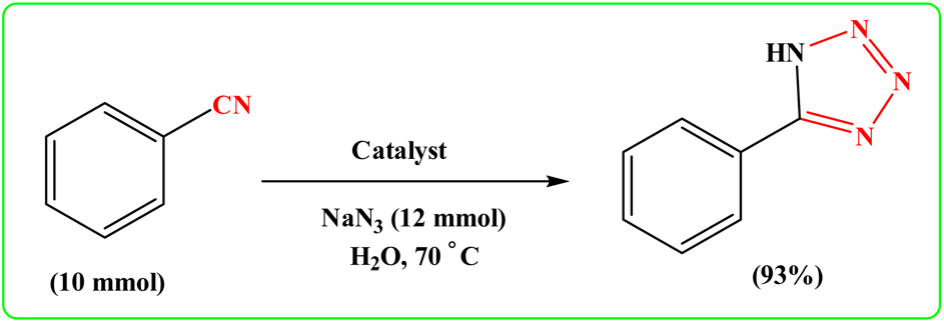
Large-scale synthesis of 5-phenyl-1*H*-tetrazole.

To showcase the practical application of the tetrazole reaction and nitrobenzene reduction, both the large-scale synthesis and the catalyst recycling experiment were performed. As shown in Scheme 4 for synthesis of aniline, a large-scale reaction was performed with nitrobenzene (10.0 mmol), NaBH_4_ (1.5 mmol), CoFe_2_O_4_@HPECG/Pr-SO_3_H·Cu(ii) (250 mg), in H_2_O (40 mL). The aniline was formed with minimal loss of yield (90%). In addition, a large-scale experiment for the synthesis of tetrazole was performed with NaN_3_ (12 mmol), benzonitrile (10 mmol), CoFe_2_O_4_@HPECG/Pr-SO_3_H·Cu(ii) (400 mg), and H_2_O (30 mL), the 5-phenyl-1*H*-tetrazole was formed with minimal loss of yield (93%) (Scheme 5). As anticipated, that the methodology is an economic and practical process for the preparation of various aniline and tetrazole products.

**Fig. 9:**
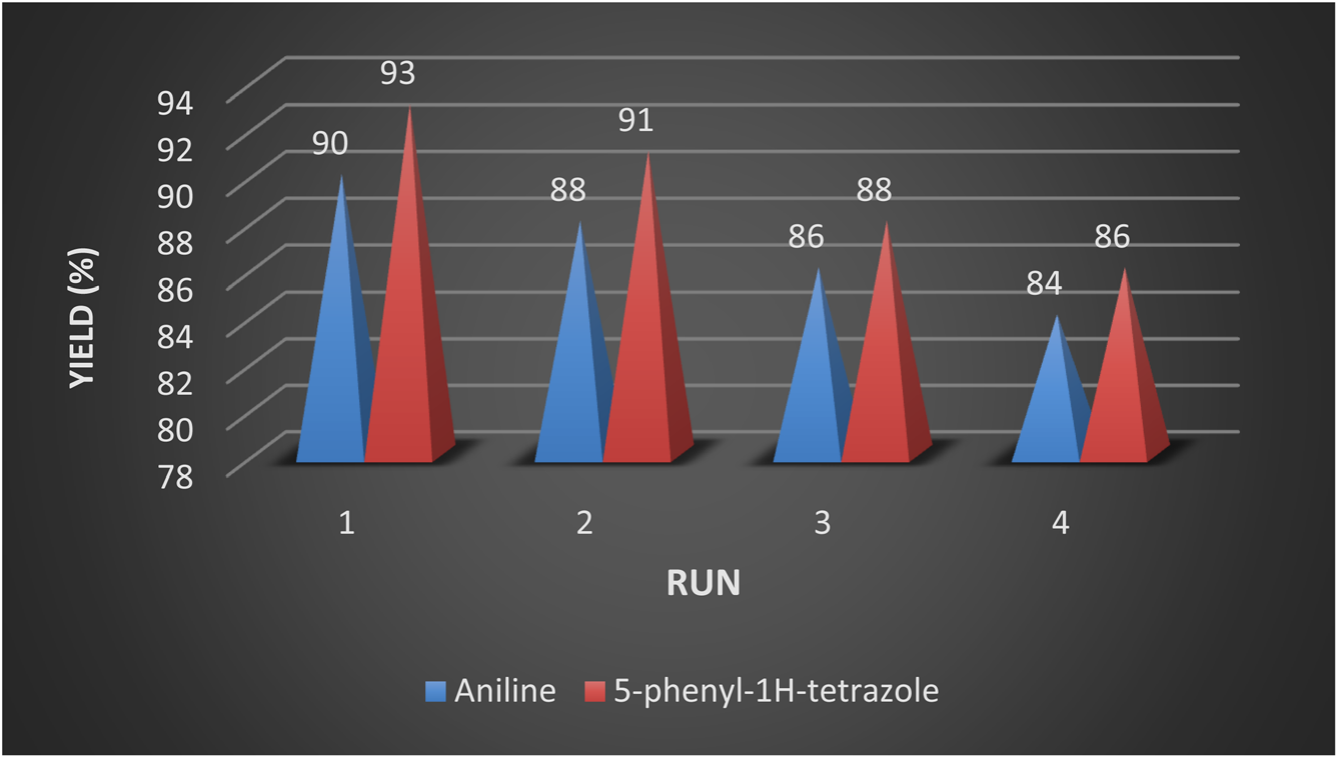
Large-scale recycling experiments for model reactions.

After completion of the reaction, the catalyst was separated from the reaction mixture by a magnet, washing and drying, then reused for the next catalytic cycle. As shown in Fig. 9, the catalyst can be reused at least 4 times with essentially no significant loss of reactivity.

## 4. Conclusions

In this research we successfully reported a green protocol for the *in situ* creation of ultrafine Cu(ii) metal immobilized on the surface of HPEC, modified by a CoFe_2_O_4_/Pr-SO_3_H magnetic nanocomposite. Then the heterogeneous catalyst's structure and composition were thoroughly analyzed using various techniques such as FT-IR, FE-SEM, VSM, ICP-OES, TGA, XRD, BET, EDX, and X-ray mapping. The catalytic performance of the CoFe_2_O_4_@HPECG/Pr-SO_3_H·Cu(ii) nanocomposite was explored in the synthesis of 5-substituted 1*H*-tetrazoles and reduction of a variety of nitro compounds under environmentally-friendly conditions. The catalyst CoFe_2_O_4_@HPECG/Pr-SO_3_H·Cu(ii), could be retrieved with a magnet from the mixture and be used 9 times without losing its effectiveness. Based on the results obtained, several key advantages of the current method can be highlighted, which can contribute to the existing methods in the literature. These include: (1) the use of a green solvent for the reaction, which aligns with environmentally friendly practices; (2) the ability to reuse the catalyst, offering both economic and environmental benefits; (3) easy separation of the catalyst and products, which simplifies the process and reduces the effort involved in purification; and (4) efficient reaction yield and timing, ensuring that the process is both effective and practical for various applications. These attributes collectively enhance the method's value and applicability in the field of chemical synthesis.

## Data availability

The datasets used and/or analyzed during the current study are available from the corresponding author on reasonable request.

## Conflicts of interest

There are no conflicts to declare.

## References

[cit1] Antenucci A., Dughera S., Renzi P. (2021). ChemSusChem.

[cit2] Tamoradi T., Ghadermazi M., Ghorbani-Choghamarani A. (2019). J. Porous Mater..

[cit3] Mahmoudi B., Rostami A., Kazemnejadi M., Hamah-Ameen B. A. (2020). Green Chem..

[cit4] Gómez-López P., Puente-Santiago A., Castro-Beltrán A., do Nascimento L. A. S., Balu A. M., Luque R., Alvarado-Beltrán C. G., Opin C. (2020). Green Sustainable Chem..

[cit5] Shokri Z., Azimi N., Moradi S., Rostami A. (2020). Appl. Organomet. Chem..

[cit6] Becker J., Manske C., Randl S. (2022). Curr. Opin. Green Sustainable Chem..

[cit7] Li C. J., Chen L. (2006). Chem. Soc. Rev..

[cit8] Ghadermazi M., Moradi S., Mozafari R. (2020). RSC Adv..

[cit9] Liu X., Liu J., Lin S., Zhao X. (2020). Mater. Today.

[cit10] Rinoldi C., Lanzi M., Fiorelli R., Nakielski P., Zembrzycki K., Kowalewski T., Urbanek O., Grippo V., Jezierska-Wozniak K., Maksymowicz W., Camposeo A., Bilewicz R., Pisignano D., Sanai N., Pierini F. (2021). Biomacromolecules.

[cit11] Zheng S. Y. , Du C., Wu Z. L., Hydrogels for Tissue Engineering and Regenerative Medicine, 2024, pp. 331–346

[cit12] Ahmed E. M. (2015). J. Adv. Res..

[cit13] Ebert B., Birdseye D., Liwanag A. J. M., Laursen T., Rennie E. A., Guo X., Catena M., Rautengarten C., Stonebloom S. H., Gluza P., Pidatala V. R., Andersen M. C. F., Cheetamun R., Mortimer J. C., Heazlewood J. L., Bacic A., Clausen M. H., Willats W. G. T., Scheller H. V. (2018). Plant Cell Physiol..

[cit14] Sahiner N. (2013). Prog. Polym. Sci..

[cit15] Sharma G., Thakur B., Naushad M., Kumar A., Stadler F. J., Alfadul S. M., Mola G. T. (2018). Environ. Chem. Lett..

[cit16] Seidi F., Yazdi M. K., Jouyandeh M., Habibzadeh S., Munir M. T., Vahabi H., Bagheri B., Rabiee N., Zarrintaj P., Saeb M. R. (2022). Carbohydr. Polym..

[cit17] Sandford P. A. , Baird J., Polysaccharides, 1983, pp. 411–490

[cit18] Bertolino V., Cavallaro G., Milioto S., Lazzara G. (2020). Carbohydr. Polym..

[cit19] Mohnen D. (2008). Curr. Opin. Plant Biol..

[cit20] Harholt J., Suttangkakul A., Vibe Scheller H. (2010). Plant Physiol..

[cit21] Thakur B. R., Singh R. K., Handa A. K. (1997). Crit. Rev. Food Sci. Nutr..

[cit22] Moradi S., Mozafari R., Ghadermazi M. (2024). J. Inorg. Organomet. Polym. Mater..

[cit23] Mozafari R., Ghadermazi M. (2020). RSC Adv..

[cit24] Mazzola L. (2003). Nat. Biotechnol..

[cit25] Rauf M., Shah S. S., Shah S. K., Shah S. N. A., Haq T. U., Shah J., Ullah A., Ahmad T., Khan Y., Aziz M. A. (2022). J. Saudi Chem. Soc..

[cit26] Mozafari R., Heidarizadeh F., Nikpour F. (2019). Mater. Sci. Eng., C.

[cit27] Zhu Q., Chua M. H., Ong P. J., Lee J. J. C., Chin K. L. O., Wang S., Kai D., Ji R., Kong J., Dong Z. (2022). Mater. Today Adv..

[cit28] ur Rehman A., Aadil M., Zulfiqar S., Agboola P. O., Shakir I., Aboud M. F. A., Haider S., Warsi M. F. (2021). Ceram. Int..

[cit29] Sorkhabi S., Ghadermazi M., Mozafari R. (2021). ChemistrySelect.

[cit30] Rossi L. M., Costa N. J., Silva F. P., Wojcieszak R. (2014). Green Chem..

[cit31] Tian S., Hu M., Xu Q., Gong W., Chen W., Yang J., Zhu Y., Chen C., He J., Liu Q. (2020). Sci. China Mater..

[cit32] Villegas V. A. R., Ramírez J. I. D. L., Guevara E. H., Sicairos S. P., Ayala L. A. H., Sanchez B. L. (2020). J. Saudi Chem. Soc..

[cit33] Deng Y. D., Wang L. J., Zhang W. H., Xu J., Gao J. J., Wang B., Fu X. Y., Han H. J., Li Z. J., Wang Y., Tian Y. S., Peng R. H., Yao Q. H. (2022). Ecotoxicol. Environ. Saf..

[cit34] Hajdu V., Muranszky G., Nagy M., Kopcsik E., Kristaly F., Fiser B., Viskolcz B., Vanyorek L. (2022). Int. J. Mol. Sci..

[cit35] Hajdu V., Muranszky G., Prekob A., Kristaly F., Fiser B., Lakatos J., Viskolcz B., Vanyorek L. (2022). J. Mater. Res. Technol..

[cit36] Molaei S., Tamoradi T., Ghadermazi M., Ghorbani-Choghamarani A. (2018). Microporous Mesoporous Mater..

[cit37] Tamoradi T., Ghorbani-Choghamarani A., Ghadermazi M. (2019). Solid State Sci..

[cit38] Ghorbani-Choghamarani A., Mohammadi M., Tamoradi T., Ghadermazi M. (2019). Polyhedron.

[cit39] Moradi S., Mozafari R., Ghadermazi M. (2024). Sci. Rep..

[cit40] Kumar A., Chauhan G. S. (2010). Carbohydr. Polym..

[cit41] Pantić M., Kravanja K. A., Knez Ž., Novak Z. (2021). Polym.

[cit42] Sharma U., Kumar N., Verma P. K., Kumar V., Singh B. (2012). Green Chem..

[cit43] Emam H. E., Saad N. M., Abdallah A. E. M., Ahmed H. B. (2020). Int. J. Biol. Macromol..

[cit44] Patra A. K., Vo N. T., Kim D. (2017). Appl. Catal., A.

[cit45] Azaroon M., Kiasat A. R. (2018). Catal. Lett..

[cit46] Karami S., Zeynizadeh B., Shokri Z. (2018). Cellulose.

[cit47] Singh A., Agarwal A. (2023). Mol. Catal..

[cit48] Nasrollahzadeh M., Motahharifar N., Nezafat Z., Shokouhimehr M. (2021). J. Mol. Liq..

[cit49] Molaei S., Ghadermazi M. (2024). Inorg. Chem. Commun..

[cit50] Jani M. A., Bahrami K. (2020). Appl. Organomet. Chem..

